# Molecular–clinical characteristics and treatment outcomes in 163 metastatic colorectal neuroendocrine carcinomas with a comparison to colorectal adenocarcinomas

**DOI:** 10.1002/ijc.70367

**Published:** 2026-02-05

**Authors:** Siren Morken, Seppo W. Langer, Geir Olav Hjortland, Anna Sundlöv, Eva Hofsli, Morten Ladekarl, Elizaveta Tabaksblat, Lene Weber Vestermark, Johanna Svensson, Ulrich Knigge, Luís Nunes, Bengt Glimelius, Per Pfeiffer, Kristine Aasebø, Jörg Assmus, Erik Vassella, Inger Marie Bowitz Lothe, Anne Couvelard, Aurel Perren, Stian Knappskog, Halfdan Sorbye

**Affiliations:** ^1^ Cancer Clinic Haukeland University Hospital Bergen Norway; ^2^ Department of Clinical Science University of Bergen Bergen Norway; ^3^ Department of Oncology Rigshospitalet Copenhagen Denmark; ^4^ Department of Clinical Medicine University of Copenhagen Copenhagen Denmark; ^5^ Department of Oncology Oslo University Hospital Oslo Norway; ^6^ Division of Oncology, Department of Clinical Sciences Lund Lund University Lund Sweden; ^7^ Department of Oncology St. Olav's Hospital Trondheim Norway; ^8^ Department of Clinical and Molecular Medicine Norwegian University of Science and Technology Trondheim Norway; ^9^ Department of Oncology Aarhus University Hospital Aarhus Denmark; ^10^ Department of Oncology, Clinical Cancer Research Center, Aalborg University Hospital Aalborg University Aalborg Denmark; ^11^ Department of Clinical Medicine Aalborg University Aalborg Denmark; ^12^ Department of Oncology University Hospital of Southern Denmark Esbjerg Denmark; ^13^ Department of Oncology Sahlgrenska University Hospital Gothenburg Sweden; ^14^ Department of Surgery, ENETS Center of Excellence Copenhagen University Hospital, Rigshospitalet Copenhagen Denmark; ^15^ Department of Clinical Endocrinology, ENETS Center of Excellence Copenhagen University Hospital, Rigshospitalet Copenhagen Denmark; ^16^ Department of Immunology, Genetics and Pathology Uppsala University Uppsala Sweden; ^17^ Department of Molecular Oncology, Institute for Cancer Research Oslo University Hospital Oslo Norway; ^18^ Department of Oncology Odense University Hospital Odense Denmark; ^19^ Centre for Clinical Research Haukeland University Hospital Bergen Norway; ^20^ Institute of Tissue Medicine and Pathology University of Bern Bern Switzerland; ^21^ Department of Pathology Oslo University Hospital Oslo Norway; ^22^ Department of Pathology Bichat Hospital AP‐HP and Paris Cité University Paris France

**Keywords:** adenocarcinoma, chemotherapy outcomes, colorectal, molecular alterations, neuroendocrine carcinoma

## Abstract

There is limited data regarding the rare and aggressive colorectal neuroendocrine carcinoma (CR‐NEC). In this large prospective study, molecular–clinical characteristics and treatment outcomes following palliative chemotherapy are reported for 163 metastatic CR‐NEC patients, with a comparison to a population‐based prospective cohort of 263 metastatic colorectal adenocarcinoma (CR‐AC) patients. Eighty‐three percent of CR‐NEC received first‐line platinum‐etoposide, while 98% of CR‐AC patients received first‐line fluorouracil‐based chemotherapy. Disease control rate across all first‐line regimens in CR‐NEC and CR‐AC was 43% vs. 74%, immediate progressive disease 46% vs. 15%, progression‐free survival 2.4 months (m) (95% CI 2.1–3.3) vs. 7.7 m (95% CI 6.9–8.5), and overall survival 6.7 m (95% CI 5.6–8.8) vs. 16.8 m (95% CI 13.7–20.3), all, *p* < .001. CR‐NEC more often had synchronous metastases, worse performance status, and symptom burden at treatment initiation than CR‐AC (all, *p* < .001). Two‐year survival was 9% vs. 37% in CR‐NEC and CR‐AC (*p* < .001). *BRAF* mutations were frequent in CR‐NEC and CR‐AC (26% vs. 20%, *p* = .153) and associated with shorter OS in CR‐NEC and CR‐AC (*p* = .025 and *p* = .003). *KRAS* mutations were less frequent in CR‐NEC than CR‐AC (34% vs. 45%, *p* = .041), but only associated with shorter OS in rectal NEC (*p* = .04). The frequencies of *APC* and *TP53* mutations were similar between the cohorts and did not impact survival. Metastatic CR‐NEC and CR‐AC are clinically distinct, with NEC demonstrating more aggressive features, limited treatment effect, and worse prognosis. Although they share important driver mutations, the underlying reason for their marked clinical differences remains unclear.

## Graphical abstract

What's new?

Colorectal neuroendocrine carcinoma (CR‐NEC) is a rare digestive neuroendocrine neoplasm that frequently exhibits an adenocarcinoma component. Nonetheless, the underlying biology of CR‐NEC remains poorly understood. This study examined molecular and clinical features and palliative chemotherapy outcomes of metastatic CR‐NEC, with comparison to metastatic colorectal adenocarcinoma (CR‐AC). Metastatic CR‐NEC and metastatic CR‐AC exhibited notable clinical differences. In particular, metastatic CR‐NEC had an extremely poor prognosis, a high rate of immediate disease progression, and short progression‐free survival. Notably, *BRAF* and *KRAS* mutations were associated with poor prognosis. Despite sharing key driver mutations, the reason for clinical differences between CR‐NEC and CR‐AC remains unclear.

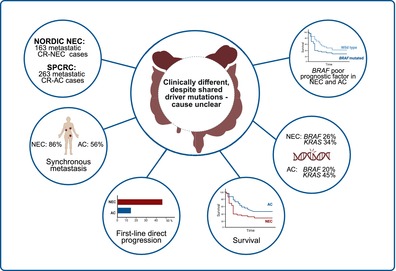

**Abbreviations**
ALP: alkaline phosphatase
CAPTEM: capecitabine and temozolomide
CI: confidence interval
CR: complete response
CR‐AC: colorectal adenocarcinoma
CR‐NEC: colorectal neuroendocrine carcinoma
DCR: disease control rate
EP: platinum and etoposide
FLOX: fluorouracil and oxaliplatin
HG‐NEN: high‐grade neuroendocrine neoplasm
ICI: immune checkpoint inhibitors
LC: large cell
m: months
MiNEN: mixed neuroendocrine‐non‐neuroendocrine neoplasm
MSI‐H: microsatellite instability‐high
NCI: National Cancer Institute
NET: neuroendocrine tumor
NGS: next‐generation sequencing
OS: overall survival
PD: progressive disease
PFS: progression‐free survival
PR: partial response
PS: performance status
RR: response rate
SC: small cell
SD: stable disease
SPCRC: Scandinavian Prospective Colorectal Cancer Registration
TMB: tumor mutation burden
WHO: World Health Organization

## 1 INTRODUCTION

Colorectal neuroendocrine carcinoma (CR‐NEC) is a rare subgroup of digestive neuroendocrine neoplasms.^1^, ^2^ These tumors are poorly differentiated, with a high proliferation rate and a poor prognosis. Most digestive NEC patients have advanced/metastatic disease at diagnosis (69–79%),^1^, ^3^, ^4^ and recurrence after initial localized disease is frequent.^5^ Therefore, palliative chemotherapy is the main treatment option for metastatic CR‐NEC.

While colon and rectum represent the most common primary sites of digestive NEC in Western countries, CR‐NEC accounts for less than 1% of all colorectal cancers.^1^, ^6^, ^7^ CR‐NEC frequently has genetic alterations in well‐known driver genes for colorectal adenocarcinoma (CR‐AC)^8^, ^9^, ^10^, ^11^, ^12^ and many CR‐NEC cases have an adenocarcinoma component. If each component accounts for at least 30% of the tumor, it is classified as a mixed neuroendocrine‐non‐neuroendocrine neoplasm (MiNEN).^13^ Their frequent co‐existence and shared molecular landscape raise the hypothesis that CR‐NEC and CR‐AC have a common clonal origin.^8^, ^13^, ^14^ Although no direct comparisons have been made, the clinical differences between CR‐NEC and CR‐AC appear to be substantial, with NEC presenting more frequently with synchronous metastasis, a much higher recurrence rate after radical surgery, and a shorter survival following palliative chemotherapy.^5^, ^15^, ^16^

The current first‐line treatment strategy, platinum combined with etoposide (EP), was historically extrapolated from the more common small‐cell lung cancer, as efficacy data on advanced digestive NEC were limited.^17^, ^18^, ^19^ However, the expected survival in patients with advanced digestive NEC receiving this regimen is less than a year. The prognosis for patients with CR‐NEC is particularly poor, with a median progression‐free survival (PFS) of 3 months (m) and a median overall survival (OS) of 8 m.^16^, ^20^, ^21^ Disease progression before or at the first response assessment, (immediate disease progression), is experienced by over half of CR‐NEC patients,^16^ challenging the rationale behind the current treatment approach.

Alternative strategies with CR‐AC regimens (fluorouracil combined with oxaliplatin and/or irinotecan) are increasingly used in CR‐NEC, especially in a second‐line setting.^19^ However, whether this approach is more beneficial than first‐line EP is under investigation.^22^ While treatment with *BRAF* inhibitors is well established for *BRAF* V600E‐mutated CR‐AC,^23^ and immune checkpoint inhibitors (ICI) for microsatellite instability‐high (MSI‐H)/mismatch repair‐deficient metastatic CR‐AC patients,^24^ their role has yet to be determined in metastatic CR‐NEC.

The underlying biological reasons for CR‐NEC having one of the poorest prognoses among all cancers remain unclear. In this study, we present novel prospective population‐based data on the molecular and clinical characteristics, as well as treatment outcomes following palliative chemotherapy in metastatic CR‐NEC, and compare them with those of metastatic CR‐AC. This study aims to improve our understanding of CR‐NEC in the pursuit of more effective treatment options for these patients.

## 2 MATERIALS AND METHODS

### 2.1 Patients and samples

#### 2.1.1 Metastatic colorectal neuroendocrine carcinoma cohort

Patients diagnosed with high‐grade neuroendocrine neoplasm (HG‐NEN) with a digestive primary site were prospectively included in the NORDIC NEC Registry from nine Scandinavian hospitals during 2013–2017. The last follow‐up was in 2021. Each participating center provided oncological treatment to all digestive HG‐NEN patients within their respective regions.

For the present work, we drew a CR‐NEC cohort by identifying all metastatic patients with a colorectal primary who had received first‐line palliative chemotherapy. Originally, 290 colorectal cases were prospectively included in the NORDIC NEC Registry. Due to changes in the 2019 World Health Organization (WHO) classification, available slides were re‐evaluated by three experienced neuroendocrine pathologists (IMBL, AC, and AP) after study inclusion without access to clinical data. The re‐evaluation led to the exclusion of 65 patients (MiNEN, well‐differentiated neuroendocrine tumors [NET], and synaptophysin staining adenocarcinomas). Of the remaining 225 confirmed CR‐NEC cases, 195 had advanced disease. Thirty‐two patients with metastatic disease did not receive palliative chemotherapy, while 163 patients (162 metastatic and one advanced unresectable locoregional disease) received treatment and were included in our present analysis. Our CR‐NEC cohort includes patients that overlap with previously published works from our group (Elvebakken et al., 2024 (*n* = 72) and Sorbye et al., 2025 (*n* = 163)).^4^, ^25^ However, our present study investigates a pure metastatic CR‐NEC cohort receiving palliative chemotherapy, which has not been previously reported. Figure S1, Supporting Information shows a flow chart of patient selection.

Radiological response to treatment was reported according to RECIST 1.1. The disease control rate (DCR) included patients with complete response (CR), partial response (PR), and stable disease (SD). Progressive disease (PD) was confirmed either radiologically or by the treating physician based on clinical progression; in patients lacking response evaluation, NEC‐specific death within 2 months after treatment termination was categorized as clinical progression (*n* = 14). When referring to evaluable patients, those with missing or non‐evaluable responses were excluded from the analysis. Cancer pain, weight loss, and anorexia were assessed according to the National Cancer Institute (NCI) grading. To evaluate the efficacy of specific regimens following first‐line treatment, we combined data from second‐ and third‐line treatments to increase our sample size and statistical power. An individual patient could contribute data from both second and third‐line treatments.

#### 2.1.2 Metastatic colorectal adenocarcinoma cohort

Given that our metastatic CR‐NEC patients were drawn from a population‐based cohort, we selected a comparative cohort of metastatic CR‐AC cases receiving palliative chemotherapy from the population‐based Scandinavian Prospective Colorectal Cancer Registration (SPCRC). The SPCRC cohort included patients from three Scandinavian hospitals during 2003–2006. The last follow‐up was in 2014. Each participating center provided oncological treatment to all CR‐AC patients within their respective regions. Clinical and molecular data for the SPCRC cohort have been previously published.^26^, ^27^, ^28^, ^29^, ^30^

Histopathological differentiation between large‐cell CR‐NEC and adenocarcinoma can be challenging. To minimize the risk of misdiagnosed cases of CR‐NEC within the CR‐AC cohort, immunohistochemical staining for the neuroendocrine marker synaptophysin was performed. Negative synaptophysin staining (here defined as nuclear fraction <1%) makes the diagnosis of CR‐NEC unlikely. From the initial cohort of 796 patients, 453 cases with available tissue microarrays were stained, and 424 synaptophysin‐negative cases were identified. Of these, 263 received first‐line palliative chemotherapy and were included in the present comparative analysis with the CR‐NEC cohort (Figure S1).

### 2.2 Molecular analysis

*BRAF*, *KRAS*, *APC*, *TP53*, *RB1*, and MSI status were collected using multiple methods. In the CR‐NEC cohort, data were obtained from previous targeted sequencing of a custom‐designed 360‐cancer gene panel (*n* = 78),^8^ plasma‐based sequencing of an Illumina TSO500 ctDNA panel (*n* = 23), and local routine testing for *BRAF* and *KRAS* status (*n* = 21). In the CR‐AC cohort, data were obtained from previous sequencing of a custom‐designed Ampliseq hotspot panel (*n* = 234),^26^ *BRAF* and *KRAS* pyrosequencing (*n* = 259 and *n* = 257, respectively),^27^ and *BRAF* immunohistochemistry (*n* = 262).^28^

In both cohorts, the canonical driver mutations for *BRAF* V600E and *KRAS* codons 12–13 were covered. In discordant cases, any positive *BRAF* or *KRAS* status was considered valid. Notably, in the CR‐NEC cohort, *APC*, *TP53*, and *RB1* mutation status were assessed from targeted next‐generation sequencing (NGS) panels (covering the entire coding region of the genes), while in the CR‐AC cohort, these genes were assessed from a hotspot panel (targeting predefined hotspot regions of the genes). Therefore, we limited the comparison of *APC*, *TP53*, and *RB1* mutation frequencies between CR‐NEC and CR‐AC to those mutations covered by the hotspot panel.

Details on the different methods applied, identification of driver mutations, and coverage of the hotspot panel are provided in Tables S1–S4. The sequencing coverage and quality statistics for each sample generated by NGS are summarized in Table S5.

### 2.3 Statistics

Descriptive statistics were used for cohort characterization. Categorical variables were compared using Fisher's exact or chi‐square test and continuous variables were compared using Mann–Whitney test. Kaplan–Meier survival with confidence intervals (CI) were calculated using the default log‐transformation method. For small sample sizes, CI was calculated with the plain method to prevent missing values. Comparisons of survival between groups were done using the log‐rank and Breslow test. Cox analyses for median PFS and median OS were conducted in several steps. The univariable model was estimated for each variable, and the multivariable model was estimated for all variables. Variables with a *p*‐value <.1 in at least one of the models, alongside clinically relevant variables (age and sex), were added to the final model. Additionally, we estimated a multivariable model containing all blood‐related variables to select one representative variable, aiming to reduce the number of highly correlated variables in the final model. Two‐sided *p*‐values <.05 were considered statistically significant. Statistical analyses were performed using IBM SPSS Statistics v. 26.0 and 30.0 (IBM Corp, Armonk, NY) and R version 4.4.1 and 4.5.1 with the survival package version 3.4.3. Figures were created in R.

## 3 RESULTS

### 3.1 Patient and pathological characteristics of metastatic colorectal NEC

Among the 195 confirmed CR‐NEC cases with metastatic disease, 32 patients did not receive palliative chemotherapy, most frequently due to poor performance status (PS) (*n* = 17) and high age (*n* = 5) (Figure S1). These patients had a short OS of 2.6 m (95% CI 1.5–7.0). The remaining 163 patients receiving palliative chemotherapy were included in the present analysis, and their baseline characteristics are summarized in Table 1. The median follow‐up time for the cohort was 73 m (95% CI 53–94), and at the last observation time, 159 (97%) patients had died. Disease‐related death was 96%, including three patients registered with chemotherapy toxicity as the immediate cause of death. Synchronous metastasis was present in 140 patients (86%), while 22 patients (13%) had metachronous metastatic disease, and one patient had a local unresectable recurrence. The primary tumor sites were right colon (*n* = 62), left colon (*n* = 22), and rectum (*n* = 79). The median Ki‐67 index was 90% (range 23–100%), with only 13 patients having a Ki‐67 ≤55% (Table S6). Large cell (LC) morphology was most common in the colon (76%), while LC and small cell (SC) were equally distributed in patients with a rectal primary (50%). Seventy‐three patients were smokers or ex‐smokers (51%), equally common between colon/rectum (53% vs. 49%) and LC/SC (50% vs. 54%). Rectal cases were more often associated with bone metastasis (colon 8% vs. rectum 21%, *p* = .02). Only one patient had brain metastasis at baseline, but 10% developed brain metastasis during the disease course; the majority of whom had a rectal primary tumor (*n* = 11/15, colon vs. rectum *p* = .059) and an SC morphology (*n* = 10/15, *p* = .011). Twelve patients had a documented prior history of colorectal cancer (anorectal NET *n* = 1 and adenocarcinoma *n* = 11).

**TABLE 1:** Comparison of baseline characteristics, response rates, and survival outcomes between 163 metastatic colorectal neuroendocrine carcinoma (CR‐NEC) and 263 metastatic colorectal adenocarcinoma (CR‐AC) patients receiving first‐line chemotherapy.

	CR‐NEC		CR‐AC		*p*‐value^c^
	Valid cases	*N* (%)	Valid cases	*N* (%)	
Age in years, median (range)	163	67.7 (29.6–87.7)	262	64.4 (23.5–85.1)	.**027**
>75 years		34 (21)		40 (15)	.139
Male	163	94 (58)	263	128 (49)	.071
Primary site	163		263		
Colon right		62 (38)		89 (34)	
Colon left		22 (14)		102 (39)	**<.001**
Rectum		79 (48)		69 (26)	**<.001**
Multiple		‐		3 (1)	
Smoker‐prior smoker^a^	143	73 (51)	237	61 (26)	**<.001**
Performance status^a^	159		263		
0		50 (31)		134 (51)	**<.001**
1		73 (46)		85 (32)	
2		25 (16)		37 (14)	
3		11 (7)		7 (3)	
Ki‐67, median (range)	163	90 (23–100)	263	‐	
≤50%		13 (8)		33 (13)	.139
>50–75%		30 (18)		85 (32)	.**002**
>75%		120 (74)		145 (55)	**<.001**
Cell type	163			‐	
Large cell		99 (61)		‐	
Small cell		56 (34)		‐	
Unspecified		8 (5)		‐	
Primary tumor resected	163	58 (36)	263	240 (91)	**<.001**
Secondary radical metastatic surgery	163	3 (2)	262	30 (11)	**<.001**
Adjuvant/neo‐adjuvant chemotherapy	163	18 (11)	263	53 (20)	
Synchronous metastasis	163	140 (86)	263	148 (56)	**<.001**
Sites of metastasis	163		263		
Liver		129 (79)		178 (68)	.**010**
Lymph nodes		61 (37)		76 (29)	
Lung		29 (18)		68 (26)	
Peritoneum		6 (4)		54 (20)	**<.001**
Peritoneum in colon primaries	84	5 (6)	191	46 (24)	**<.001**
Peritoneum in rectal primaries	79	1 (1)	69	7 (10)	.**025**
Bone		24 (15)		13 (5)	**<.001**
Symptom burden (all NCI grades)^a^					
Pain	160	95 (59)	261	96 (37)	**<.001**
Weight loss ≥5% last 3 months	156	67 (43)	255	96 (38)	.286
Anorexia	157	70 (45)	256	69 (27)	**<.001**
Development of brain metastasis^a^	155	15 (10)	262	21 (8)	
FDG‐PET uptake^a^	58	58 (100)		‐	
SRI + Octreoscan > liver^a^, ^b^	36	7 (19)		‐	
CgA serum > UNL^a^	122	58 (47)		‐	
NSE > UNL^a^	82	59 (72)		‐	
LDH > UNL^a^	147	69 (47)	239	106 (44)	.620
ALP > UNL^a^	158	89 (56)	249	137 (55)	.796
Platelets >400 × 10^9^/L^a^	160	42 (26)	248	56 (24)	.349
WBC >10 × 10^9^/L^a^	160	53 (33)	257	52 (20)	.**003**

Abbreviations: ALP, alkaline phosphatase; CgA, chromogranin A; FDG‐PET, fluorodeoxyglucose‐positron emission tomography; Ga‐PET, gallium‐positron emission tomography; LDH, lactate dehydrogenase; NCI, National Cancer Institute; NSE, neuron specific enolase; UNL, upper normal limit; WBC, white blood cells.

^a^ Percentage as a fraction of examined patients.

^b^ 68Ga‐DOTATATE PET/CT (*n* = 17), 111In‐octreotide SPECT (*n* = 26), both imaging modalities (*n* = 7, with same result).

^c^ Continuous variables were compared using the Mann–Whitney *U* test; categorical variables were compared using Fischer's exact test when the sample size was <5, and otherwise by chi‐square test. Bold values indicate statistical significance (*p* <.05).

#### 3.1.1 First‐line chemotherapy

**FIGURE 1:**
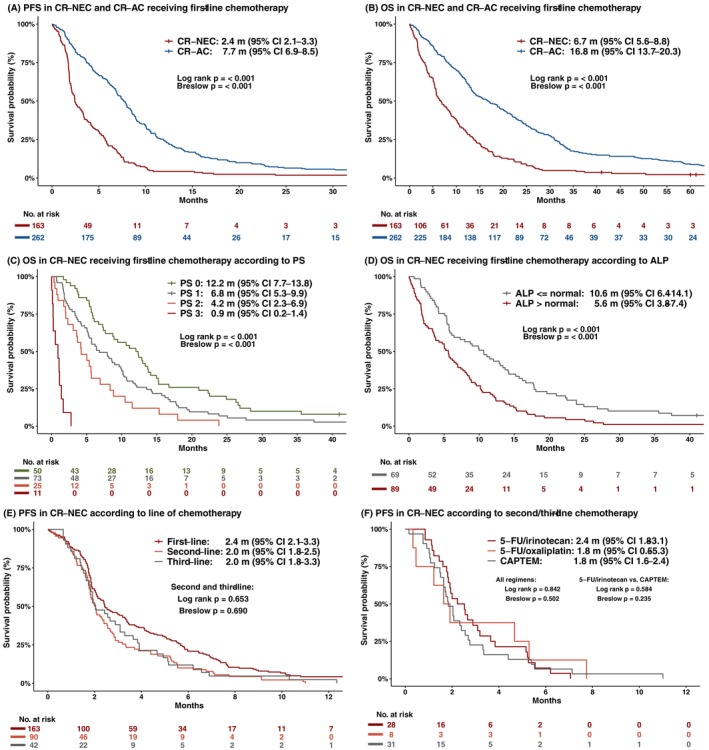
Median progression‐free survival (PFS) (A) and median overall survival (OS) (B) in metastatic colorectal neuroendocrine carcinoma (CR‐NEC) and metastatic colorectal adenocarcinoma (CR‐AC) patients receiving first‐line palliative chemotherapy. Median OS in metastatic CR‐NEC according to (C) performance status (PS) and (D) alkaline phosphatase (ALP). PFS in metastatic CR‐NEC according to (E) line of chemotherapy and (F) second and third‐line chemotherapy regimens.

In the 163 CR‐NEC patients receiving first‐line treatment, the response rate (RR) was 24% (CR 2% and PR 22%), and 19% achieved SD, while 46% had PD as best overall response (Table 2). The median PFS was 2.4 m (95% CI 2.1–3.3), and the median OS was 6.7 m (95% CI 5.6–8.8) (Figure 1A,B), with no difference according to primary site (Figure S2A,B). The 2‐ and 5‐year survival rates were 9% and 2%, respectively. Comparing cases with CR/PR to SD, no difference in PFS (6.4 m vs. 5.5 m, *p* = .53) or OS (13.0 m vs. 12.2 m, *p* = .79) was observed.

**TABLE 2:** Response rates and survival outcomes in metastatic colorectal NEC patients receiving first‐, second‐, and third‐line palliative chemotherapy.

	Valid cases	CR/PR, *N* (%)	SD, *N* (%)	PD^a^, *N* (%)	NE/NA, *N* (%)	Median PFS (95% CI), months	*p*‐value^g^	Median OS (95% CI), months	*p*‐value^g^
First‐line	163	39 (24)	31 (19)	75 (46)	18 (11)	2.4 (2.1–3.3)		6.7 (5.6–8.8)	
Colon right	62	12 (19)	16 (26)	28 (45)	6 (10)	2.7 (2.1–3.7)	.958	7.2 (5.6–9.9)	.485
Colon left	22	4 (18)	6 (27)	11 (50)	1 (5)	2.0 (1.6–5.6)		4.5 (2.8–11.5)	
Rectum	79	23 (29)	9 (11)	36 (46)	11 (14)	2.4 (1.9–3.8)		6.4 (5.4–10.5)	
Regimens	163								
Platinum^b^/etoposide	136	33 (24)	26 (19)	63 (47)	14 (10)	2.4 (2.0–3.3)	.453	6.8 (5.4–8.8)	.917
Fluorouracil‐based^c^	11	4 (37)	2 (18)	3 (27)	2 (18)	4.4 (0.9–5.2)		5.6 (3.9–14.1)	
CAPTEM	6	0	1 (17)	3 (50)	2 (33)	2.6 (2.4–4.1)		7.2 (4.2–23.8)	
Other^d^	10	2 (20)	2 (20)	6 (60)	0	2.8 (1.9–4.3)		12.5 (5.2–16.2)	
Ki‐67	163						.**002**		.916
≤55%	13	0	2 (15)	10 (77)	1 (8)	1.9 (1.0–2.5)		5.1 (1.6–11.9)	
>55%	150	39 (26)	29 (19)	65 (44)	17 (11)	2.6 (2.1–3.4)		6.9 (5.7–8.7)	
PS	159						**<.001**		**<.001**
0	50	17 (34)	12 (24)	17 (34)	4 (8)	4.2 (2.5–5.8)		12.2 (7.7–13.8)	
1	73	17 (23)	14 (19)	34 (47)	8 (11)	2.4 (2.1–3.3)		6.8 (5.3–9.9)	
2	25	4 (16)	2 (8)	15 (60)	4 (16)	1.8 (1.4–3.3)		4.2 (2.3–6.9)	
3	11	0	1 (9)	9 (82)	1 (9)	0.9 (0.2–1.3)		0.9 (0.2–1.4)	
Comparison of lines							.653		.532
Second‐line	90	13 (14)	11 (12)	61 (68)	5 (6)	2.0 (1.8–2.5)		4.6 (3.6–7.4)	
Third‐line	42	4 (9)	11 (27)	23 (55)	4 (9)	2.0 (1.8–3.3)		4.9 (3.1–6.4)	
Second‐ and third‐line combined									
Irinotecan‐based^e^	28	4 (14)	6 (22)	16 (57)	2 (7)	2.4 (1.8–3.1)	.842	‐	
Oxaliplatin‐based^f^	8	2 (25)	2 (25)	4 (50)	0	1.8 (0.5–5.3)		‐	
CAPTEM	31	5 (16)	2 (6)	21 (68)	3 (10)	1.8 (1.6–2.4)		‐	

Abbreviations: CAPTEM, capecitabine/temozolomide; CI, confidence interval; CR, complete response; NE/NA, not evaluable/not assessed; NEC, neuroendocrine carcinoma; OS, median overall survival; PD, progressive disease; PFS, median progression‐free survival; PR, partial response; PS, performance status; SD, stable disease.

^a^ Radiologic (RECIST) and clinical progressive disease.

^b^ Cisplatin *n* = 23, carboplatin *n* = 113.

^c^ Fluorouracil/oxaliplatin *n* = 1, FLOX *n* = 4, FOLFOX *n* = 2, FOLFIRINOX *n* = 2, FOLFOXIRI *n* = 2.

^d^ Capecitabine *n* = 3, everolimus/temozolomide *n* = 2, temozolomide *n* = 2, 5‐FU *n* = 1, ACO *n* = 1, etoposide *n* = 1.

^e^ Irinotecan‐based regimes: FLIRI *n* = 11, FOLFIRI *n* = 15, Irinotecan/Capecitabine *n* = 1, IRIS *n* = 1.

^f^ Oxaliplatin‐based regimes: Oxaliplatin/Fluorouracil *n* = 2, FLOX *n* = 3, FOLFOX *n* = 3.

^g^ Categorical variables were compared using Fischer's exact test when the sample size was <5, and otherwise by chi‐square test. Bold values indicate statistical significance (*p*<.05).

Platinum (cisplatin *n* = 23/carboplatin *n* = 113) combined with etoposide (EP) was given to 136/163 (83%) patients. The median number of treatment cycles per patient was three (range 1–11). The DCR on first‐line EP was 43%, with PFS 2.4 m, and OS 6.8 m. Impaired PS negatively influenced the DCR (PS 0: 58%, PS 1: 42%, PS 2: 24%, and PS 3: 1%). None of the nine patients with a Ki‐67 ≤55% who received first‐line EP achieved disease control. Eleven patients received a fluorouracil‐based combination regimen as first‐line treatment with DCR 55%, PFS 4.4 m (95% CI 0.9–5.2), and OS 5.6 m (95% CI 3.9–14.1).

In the whole cohort, increasingly impaired PS was associated with shorter PFS and OS (*p* < .001; Figure 1C), with PS 0 having OS 12.2 m compared to 4.2 m in patients with PS 2. Patients with metastatic liver involvement had a shorter OS than those with metastases confined only to non‐hepatic sites (5.7 m vs. 11.5 m, *p* = .002). Moreover, in a multivariable analysis of clinical baseline characteristics, poor PS was a strong prognostic factor for PFS and OS, while elevated alkaline phosphatase (ALP) was strongly associated with short OS (Figure 1D and Figures S3 and S4). Higher Ki‐67 (continuous variable) had a small (regarding hazard ratio) but statistically significant association with improved PFS (*p* < .001) and OS (*p* = .019). A detailed overview of the univariable and multivariable regression analysis is found in Table S7.

#### 3.1.2 Second‐line chemotherapy and beyond

Ninety patients received second‐line chemotherapy (Table 2): capecitabine and temozolomide (CAPTEM) 33%, other fluorouracil‐based combinations 30%, EP 9%, and other regimens 28%. Second‐line RR across all regimens was 14%, SD 12%, and PD 68%. Forty‐three of 75 patients who experienced immediate first‐line PD went on to receive second‐line treatment and achieved a RR of 14%, SD 9%, and PD 72%. Less than half (*n* = 42) of patients receiving second‐line treatment received third‐line treatment, with RR 9%, SD 26%, and PD 55%. PFS following second and third‐line treatments was similar and only 2.0 m (Figure 1E), while OS was 4.6 m and OS 4.9 m from treatment initiation, respectively. Treatment details for second and later lines of therapies are summarized in Table S8.

To evaluate the efficacy of specific regimens, we combined second‐ and third‐line treatment data, as outcome results were similar. PFS was short and showed no statistical difference between irinotecan‐based treatment (2.4 m, 95% CI 1.8–3.1), oxaliplatin‐based treatment (1.8 m, 95% CI 0.5–5.3), and CAPTEM (1.8 m, 95% CI 1.6–2.4) (Figure 1F). In all three treatment groups, PD was recorded in more than 50% of the patients.

#### 3.1.3 Molecular characteristics

**FIGURE 2:**
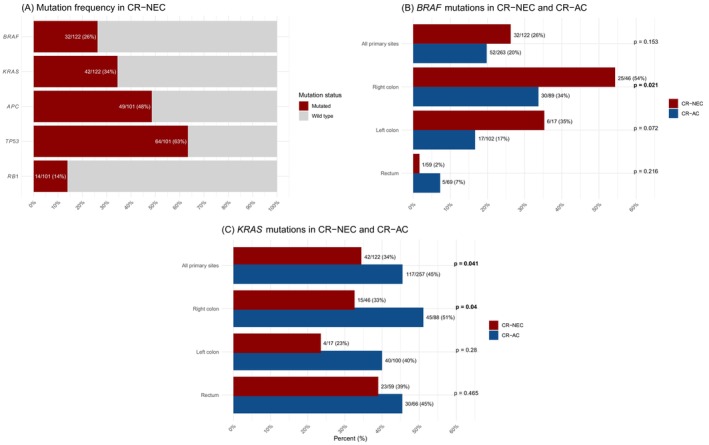
Frequency of selected driver mutations in metastatic colorectal neuroendocrine carcinoma (CR‐NEC) (A). Frequencies of *BRAF* mutations (B) and *KRAS* mutations (C) in metastatic CR‐NEC and metastatic colorectal adenocarcinoma (CR‐AC).

**FIGURE 3:**
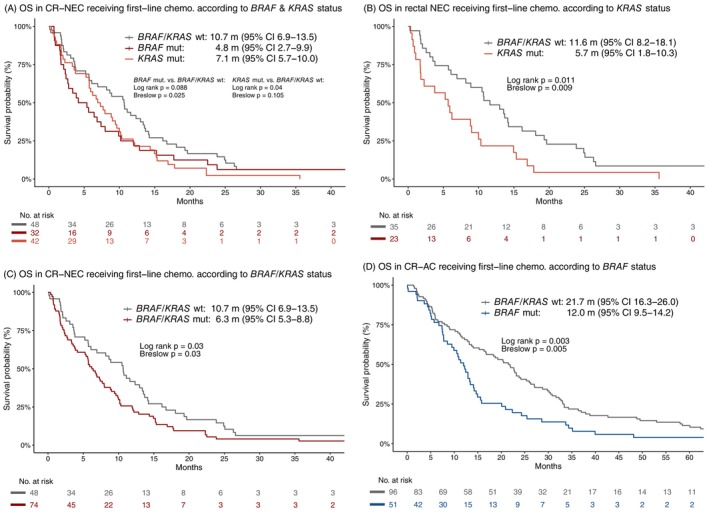
Median overall survival (OS) in metastatic colorectal neuroendocrine carcinoma (CR‐NEC) receiving first‐line palliative chemotherapy according to (A) *BRAF* and *KRAS* mutation status across all primary sites, (B) *KRAS* status in rectal NEC and (C) *BRAF*/*KRAS* status across all primary sites. Median OS in metastatic colorectal adenocarcinoma (CR‐AC) according to *BRAF* status across all primary sites (D).

Mutational data for *BRAF*, *KRAS*, *APC*, *TP53*, and *RB1* were assessed. Among patients with available *BRAF* status, *BRAF* mutation was found in 26% (32/122) (Figure 2A), occurring most frequently in the right colon (54%) and only in one rectal primary (Figure 2B). Given the functional relationship between *BRAF* and *KRAS*, survival outcomes were compared to patients having a *BRAF/KRAS* double wild‐type (wt.) status. Driver mutations in *BRAF* and *KRAS* were mutually exclusive in the CR‐NEC cohort. *BRAF*‐mutated cases had a significantly shorter OS than double wt. (4.8 m vs. 10.7 m, Breslow *p* = .025), across all primary sites (Figure 3A). The difference did not reach significance for the primary sites individually. *KRAS* mutation was found in 34% (42/122), most frequently in rectal cases (39%) (Figure 2C). OS was significantly shorter in the *KRAS*‐mutated group compared to double wt. across all primary sites (7.1 m vs. 10.7 m, *p* = .04; Figure 3A), with the difference primarily driven by patients with a rectal primary (5.7 m vs. 11.6 m, *p* = .011; Figure 3B). No similar impact on survival was observed for right and left colon. Combining patients with mutations in either *BRAF* or *KRAS*, this group also showed a significantly shorter OS compared to double wt. across all primary sites (*p* = .03; Figure 3C). No associations were observed between *BRAF* and *KRAS* mutation status and PFS, nor did alterations in *APC*, *TP53*, and *RB1* have an impact on survival outcomes (Table S9). Due to the unequal distribution of LC and SC morphology according to primary site, we separately assessed the mutational status by cell type for colonic and rectal primaries. Only *TP53* mutations in colonic primaries were found to be significantly different between LC vs. SC (73% vs. 36%, *p* = .034). MSI‐H was observed in 4% (4/107) of the patients. Tumor mutation burden (TMB) based on the 360‐cancer gene panel was available for 61 patients. The median TMB was 3.3 per MB (range 0–32.5) and showed no impact on survival.

### 3.2 Comparison of metastatic colorectal NEC and colorectal adenocarcinoma

#### 3.2.1 Baseline clinical and pathological features

Of the 424 synaptophysin‐negative CR‐AC cases with metastatic disease, 263 patients received palliative chemotherapy and were included in our comparative analysis with the CR‐NEC cohort. A comparison of baseline characteristics for the two cohorts is summarized in Table 1. The median age in the CR‐NEC cohort was higher than in the CR‐AC cohort (68 years vs. 64 years, *p* = .027), while the proportion of patients aged ≥75 years was similar between the two cohorts (*p* = .139). Smoking history was present in 51% of CR‐NEC and 26% of CR‐AC patients (*p* < .001). Primaries in the left colon were less frequent in NEC than adenocarcinomas (14% vs. 39%, *p* < .001) and more frequent in the rectum (48% vs. 26%, *p* < .001). Proliferation rate with a Ki‐67 ≤50% was infrequent in both cohorts (CR‐NEC 8% vs. CR‐AC 13%, *p* = .139), while Ki‐67 >75% was more frequent in the CR‐NEC cohort (74% vs. 55%, *p* < .001). Synchronous (86% vs. 56%, *p* < .001), liver (79% vs. 68%, *p* = .010), and bone (15% vs. 5%, *p* < .001) metastases were more frequent in CR‐NEC, while peritoneal metastasis was less frequent in NEC than in CR‐AC (4% vs. 20%, *p* < .001). At treatment initiation, only 31% of CR‐NEC were able to carry out everyday activities without restrictions (PS 0), compared to over half (51%) of the CR‐AC patients (*p* < .001). Secondary radical metastatic surgery was performed in 2% of CR‐NEC and 11% of CR‐AC (*p* < .001). Pain and anorexia (all NCI grades) were more frequently reported at the start of palliative treatment in CR‐NEC (59% and 45%) than in CR‐AC (37% and 27%) (both *p* < .001).

#### 3.2.2 Treatment and outcome

While only 7% of the CR‐NEC cohort received first‐line fluorouracil‐based regimens, such regimens were administered to 98% (257/263) of CR‐AC patients receiving first‐line palliative chemotherapy (fluorouracil and oxaliplatin [FLOX] 52%, FLOX/cetuximab 7%, fluorouracil monotherapy 21%, fluorouracil and irinotecan 18% and irinotecan monotherapy 2%). First‐line RR for CR‐NEC compared to CR‐AC was 24% vs. 37% (*p* = .006), with a DCR of 43% vs. 74% (*p* < .001) and an immediate PD of 46% vs. 15% (*p* < .001), respectively (Table 3). Among evaluable patients, the DCR was 48% (70/145) for CR‐NEC and 83% (193/233) for CR‐AC (*p* < .001). PFS was 2.4 m in CR‐NEC and 7.7 m in CR‐AC (*p* < .001), while OS was 6.7 m and 16.8 m, respectively (*p* < .001) (Figure 1A,B). Even in patients with PS 0 at the start of palliative treatment, the OS was significantly different between CR‐NEC and CR‐AC (12.2 m vs. 23.0 m, *p* < .001). No significant associations were observed between tumor sidedness and survival in the CR‐NEC or CR‐AC cohorts (*p* = .829 and *p* = .202, respectively). While no age‐related difference in survival was observed in the CR‐NEC cohort, it was observed in the CR‐AC cohort as patients over 75 years had a significantly shorter OS than those aged 75 years or younger (*p* = .029). Two‐year survival rates were 9% vs. 37% (*p* < .001) and five‐year survival was 2% vs. 9% (*p* = .002) in CR‐NEC and CR‐AC, respectively.

**TABLE 3:** Comparison of response rates and survival outcomes between 163 metastatic colorectal neuroendocrine carcinoma (CR‐NEC) and 263 metastatic colorectal adenocarcinoma (CR‐AC) patients receiving first‐line treatment.

	CR‐NEC		CR‐AC		*p*‐value^d^
	Valid cases	*N* (%)	Valid cases	*N* (%)	
Response	163		262		
CR/PR		39 (24)		96 (37)	.**006**
SD		31 (19)		97 (37)	
DCR		70 (43)		193 (74)	**<.001**
PD^a^		75 (46)		40 (15)	**<.001**
NE/NA		18 (11)		29 (11)	
Median PFS (95% CI), months	163	2.4 (2.1–3.3)	262	7.7 (6.9–8.5)	**<.001**
Colon right		2.7 (2.1–3.7)		7.5 (6.4–8.5)	**<.001**
Colon left		2.0 (1.6–5.6)		7.4 (6.4–8.5)	**<.001**
Rectum		2.4 (1.9–3.8)		8.8 (7.0–10.4)	**<.001**
Median OS (95% CI), months	163	6.7 (5.6–8.8)	262	16.8 (13.7–20.3)	**<.001**
Colon right		7.2 (5.6–9.9)		13.5 (11.8–19.3)	**<.001**
Colon left		4.5 (2.8–11.5)		19.4 (14.1–24.3)	**<.001**
Rectum		6.4 (5.4–10.5)		18.8 (13.4–24.0)	**<.001**
According to age	163		262		
≤75 years		6.9 (5.7–9.4)		18.5 (14.2–21.9)	**<.001**
>75 years		5.1 (3.9–11.1)		10.9 (6.5–20.9)	**<.001**
According to Ki‐67	163		262		
≤75%		8.7 (6.4–12.8)		16.5 (12.8–21.9)	**<.001**
>75%		5.8 (5.4–8.3)		17.0 (13.6–21.6)	**<.001**
Two‐year survival^b^	163	15 (9)	262	96 (37)	**<.001**
Five‐year survival^b^, ^c^	162	3 (2)	262	24 (9)	.**002**

Abbreviations: CI, confidence interval; CR, complete response; DCR, disease control rate; NE/NA, not evaluable/not assessed; OS, median overall survival; PD, progressive disease; PFS, median progression‐free survival; PR, partial response; SD, stable disease.

^a^ Radiologic (RECIST) and clinical progression.

^b^ From start of first‐line palliative chemotherapy.

^c^ Percentage as a fraction of examined patients.

^d^ Categorical variables were compared using Fischer's exact test when the sample size was <5, and otherwise by chi‐square test. Bold values indicate statistical significance (*p*<.05).

#### 3.2.3 Molecular characteristics

Similar frequencies of *BRAF* mutations were observed in CR‐NEC and CR‐AC (26% vs. 20%, *p* = .153); however, significantly more often in right‐sided NEC compared to right‐sided adenocarcinomas (54% vs. 34%, *p* = .021) (Figure 2B). Consistent with CR‐NEC, *BRAF* mutation negatively impacted OS for CR‐AC across all primary sites (*p* = .003, Figure 3D). *KRAS* mutations were less frequent in CR‐NEC than in CR‐AC (34% vs. 45%, *p* = .041), especially in right‐sided disease (33% vs. 51%, *p* = .040) (Figure 2C). Unlike in CR‐NEC, *KRAS* mutation did not influence OS in CR‐AC. The frequency of *BRAF*/*KRAS* double wt. did not differ between the two cohorts (*p* = .709). MSI‐H was rare in both CR‐NEC (4%) and CR‐AC (6%). Comparative analysis of *APC*, *TP53*, and *RB1* status between the two cohorts was limited to the coverage of the hotspot panel used in the CR‐AC cohort and should be interpreted with caution. *RB1* mutation was more frequent in CR‐NEC than CR‐AC across all primary sites (*p* = .024), while no difference in frequency was observed for *APC* and *TP53* (Table S10).

## 4 DISCUSSION

Metastatic CR‐NEC is an aggressive disease with a poor prognosis, but due to its rarity, there is a considerable lack of data to guide treatment. Standard first‐line palliative treatment with EP has limited benefits and poor survival outcomes.^16^ This large prospective study on 163 patients with metastatic CR‐NEC receiving palliative chemotherapy presents novel data on treatment outcomes according to treatment line and different chemotherapy regimens, as well as the impact of important colorectal driver mutations on survival. We additionally compared our data to a prospective population‐based cohort of metastatic CR‐AC to investigate the differences and similarities between these two diseases.

CR‐NEC has an aggressive phenotype and lacks effective palliative treatment. In our study, most CR‐NEC patients received first‐line standard‐of‐care with EP (83%), and nearly half (46%) experienced no clinical benefit, with immediate disease progression on treatment. An OS of 6.7 m after the start of first‐line chemotherapy illustrates that CR‐NEC is one of our most aggressive cancers. Unfortunately, no improvement in survival was observed compared to the retrospective NORDIC NEC study conducted from 2000 to 2009.^16^ Our results highlighted the urgent need for improved treatment for these patients.

It is still unknown whether patients with CR‐NEC would benefit more from an “adenocarcinoma‐like” regimen than from EP. In our cohort, patients who received first‐line fluorouracil‐based combination regimens showed no significant difference in survival compared to those who received EP. However, the fluorouracil‐treated group was limited to 11 cases, and the results should be interpreted with caution. Although not directly comparable to our data, as CR primaries were not included, encouraging results have been reported on mFOLFIRINOX in advanced gastroenteropancreatic HG‐NEN with RR 77%, PFS 12.0 m and OS of 20.6 m.^31^ Whether mFOLFIRINOX is a better treatment option than first‐line EP will hopefully be clarified by the results from the randomized FOLFIRINEC trial.^22^

Second and third‐line treatment within the CR‐NEC cohort was heterogeneous, reflecting the current lack of standardization and evidence. All regimens were associated with poor RR, short PFS, and OS. Two recent randomized phase II studies (NET‐02 and BEVANEC trials) showed a PFS between 3 and 3.5 m for fluorouracil and irinotecan.^32^, ^33^ While the effect is modest, it probably represents the best current evidence for second‐line treatment. The addition of anti‐angiogenic agents, such as bevacizumab and ramucirumab, to second‐line chemotherapy has not been shown to be superior for CR‐NEC, although it seems promising for gastric NEC.^32^, ^34^ It has previously been described that CAPTEM should be used with caution in patients with a high proliferation rate (>55%), which is the case for most CR‐NEC patients.^16^ We observed a similar trend with the pooled first, second, and third‐line response data for CAPTEM, showing immediate PD in 78% of 37 patients.

While emerging molecular data have shown many potential targetable mutations in digestive NEC,^8^, ^35^, ^36^ no targeted therapy is currently standard of care in CR‐NEC.^19^ Our reported frequencies of *BRAF* and *KRAS* mutations (*n* = 122) and *APC*, *TP53*, and *RB1* mutations (*n* = 101) were consistent with NGS data from the AACR GENIE database, including 83 CR‐NEC patients, and with a report on 10 CR‐NEC cases.^35^ Two CR‐NEC cohorts (18 and 37 cases) have reported lower *BRAF* (7–11%) and *KRAS* (17%) mutation frequencies and higher *TP53* mutation frequency (94%)^37^, ^38^; however, unlike our study, those cohorts included all stages, MiNEN cases, and did not specify colon or rectum primary.

We found that *BRAF* mutations negatively impacted OS across all primary sites, and *KRAS* mutations were associated with worse OS in rectal NEC. While *BRAF* and *KRAS* mutations are known poor prognostic factors in CR‐AC,^39^ this is the first report in a relatively large cohort of metastatic CR‐NEC. Consistent with our results, a recent retrospective study reported an association between *KRAS* mutations and worse OS in advanced gastrointestinal NEC.^40^ Although a previous small retrospective metastatic colon NEC cohort from our group (*n* = 17) and Lee et al., reporting on 30 CR‐NEC patients (only 37% had metastatic disease at diagnosis), found no impact of *BRAF* or *KRAS* mutations on survival.^9^, ^41^

Although *BRAF* V600E inhibitors are approved treatments for metastatic CR‐AC, the benefit in CR‐NEC has so far only been demonstrated in individual case reports, and targeted treatment with *KRAS* and EGFR inhibitors remains unexplored, highlighting the need for biomarker‐driven clinical trials for CR‐NEC.^19^, ^42^ In our CR‐NEC cohort, only 4% of patients were MSI‐H, and none received ICI. The results of ICI in biomarker‐unselected advanced digestive NEC have so far shown limited efficacy.^43^, ^44^ The combination of chemotherapy and ICI in the NICE‐NEC single‐arm phase II trial (first‐line EP combined with nivolumab and followed by maintenance nivolumab) showed only one response in six colorectal HG‐NEN patients.^45^ The ongoing randomized SWOG S2012 phase II/III trial will hopefully answer whether the combination of EP and atezolizumab benefits digestive NEC.^46^ TMB was only available for a subset of patients, and the median TMB was low. For the few CR‐NEC patients with high TMB and MSI‐H, the predictive value of these biomarkers for ICI response remains uncertain.^19^ In line with previous data on digestive NEC,^16^, ^20^, ^21^ poor PS and elevated ALP were associated with a shorter OS. PS impacts treatment efficacy, and careful consideration should be given to treatment initiation in patients with poor PS. A higher proliferation rate was associated with a small but significant longer PFS, and this finding is concordant with previous reports on digestive NEC, which show that patients with a higher Ki‐67 index have better efficacy of platinum‐based chemotherapy.^16^ The small association between a higher proliferation rate and better OS is more surprising but might reflect the importance of an effective first‐line treatment. Although the association was significant in our multivariable analysis for OS, the confidence intervals border on 1.00, and the result may not be clinically relevant.

Our comparison of the metastatic CR‐NEC and CR‐AC cohorts receiving palliative chemotherapy revealed significant clinical differences between the two entities, despite the presence of shared driver mutations. A smoking history was twice as common in CR‐NEC compared to CR‐AC, suggesting that smoking may be a potential risk factor for NEC. Other studies have suggested that inflammatory bowel disease and human papillomavirus may be risk factors for CR‐NEC; however, such data are unavailable in our CR‐NEC cohort.^47^ While the most and least common tumor primary sites in NEC were the rectum and left colon, respectively, the opposite was true for adenocarcinomas. The two diseases exhibited distinct metastatic patterns, with CR‐NEC more frequently presenting with synchronous metastases and bone involvement, while peritoneal metastases were uncommon. These findings suggest that CR‐NEC disseminates much earlier than CR‐AC, reflected clinically by higher symptom burden and poorer PS in NEC patients at first‐line treatment initiation.

A marked difference in treatment efficacy was observed, with only 48% of evaluable CR‐NEC patients achieving disease control, compared to 83% of evaluable CR‐AC patients. The survival discrepancy was striking, with CR‐NEC showing a PFS of only one‐third and an OS of less than half compared to CR‐AC. Less than 1 in 10 CR‐NEC patients survived 2 years after starting palliative chemotherapy, compared to more than one‐third of CR‐AC patients. A worse PS or a higher proliferation rate cannot merely explain the markedly poorer survival outcomes observed for the CR‐NEC cohort at the start of palliative chemotherapy, as the differences between the two cohorts persisted among CR‐NEC and CR‐AC patients with PS 0 and regardless of Ki‐67 below or above 75%.

In accordance with previous literature, we demonstrate here that metastatic CR‐NEC and CR‐AC share important molecular driver alterations for colorectal tumorigenesis, including *BRAF*, *KRAS*, *APC*, and *TP53*.^8^, ^9^, ^14^, ^35^, ^48^ Interestingly, although excluded from this study, 12% of the CR‐NEC cases were reclassified as MiNEN. Their molecular similarities and frequent co‐existence indicate that CR‐NEC and CR‐AC might have a common clonal origin.^48^ While *BRAF* inhibitors and “adenocarcinoma‐like regimens” might be useful in managing CR‐NEC, other treatment strategies are likely needed for this relatively chemo‐resistant disease. The reason behind the markedly worse treatment outcome for CR‐NEC compared to CR‐AC is poorly understood. Further studies are needed to understand the mechanisms behind one of the most aggressive cancer types in the hope of improving future treatments for these patients.

### 4.1 Strengths and limitations

A major strength of this study is the prospective data collection from the NORDIC NEC and SPCRC cohort, including detailed oncological treatment data. Another strength is the pathological re‐evaluation of the CR‐NEC cohort to exclude NET, MiNEN, and synaptophysin‐staining adenocarcinomas. In this study, we included only patients with metastatic disease receiving palliative chemotherapy, resulting in two comparable cohorts. The cohorts are not comparable for patients not receiving palliative chemotherapy, as only the SPCRC cohort included metastatic patients not seen in the oncology department, identified through regional cancer registries. The SPCRC cohort included patients from 2003 to 2006, which was prior to the inclusion period of the CR‐NEC cohort. While survival outcomes for metastatic CR‐AC patients in clinical trials have substantially improved, two more recent population‐based cohorts of metastatic CR‐AC from Sweden (2010–2020) and Canada (2010–2019) have reported similar OS between 13.8–17.1 m following first‐line palliative treatment, which is comparable to our data (OS 16.8 m).^49^, ^50^ Advances in treatment for metastatic CR‐AC may have further increased the current survival differences between CR‐NEC and CR‐AC. Regarding our survival analyses, we acknowledge that the calculation of confidence intervals with the plain method for small sample sizes may underestimate the uncertainty in the survival estimates. As NGS data from the CR‐AC cohort were based on a hotspot panel, the comparison of *APC*, *TP53*, and *RB1* mutation status between the two cohorts is limited to the hotspot panel coverage, and the frequencies of these mutations are most likely underestimated. While our CR‐NEC cohort is among the largest reported, we have limited patients for our subgroup analysis on different chemotherapy regimens.

## 5 CONCLUSIONS

Metastatic CR‐NEC has a poor prognosis, a high rate of immediate disease progression, and short PFS, regardless of treatment line or chemotherapy regimen. First‐line treatment with platinum and etoposide is mainly ineffective, and there is an urgent need for new therapeutic strategies. *BRAF* and *KRAS* were frequently mutated in CR‐NEC, with *BRAF* being associated with poor prognosis across all primary sites, and *KRAS* being a poor prognostic factor in rectal cases. Metastatic CR‐NEC and CR‐AC exhibit notable clinical differences, with NEC demonstrating a much poorer outcome despite the presence of shared driver mutations. The reasons behind this markedly worse treatment outcome for CR‐NEC compared to CR‐AC are poorly understood, underscoring the need for further investigation.

## AUTHOR CONTRIBUTIONS

**Siren Morken:** Writing – original draft; formal analysis; data curation; visualization. **Seppo W. Langer:** Writing – review and editing; investigation. **Geir Olav Hjortland:** Investigation; writing – review and editing. **Anna Sundlöv:** Investigation; writing – review and editing. **Eva Hofsli:** Investigation; writing – review and editing. **Morten Ladekarl:** Investigation; writing – review and editing. **Elizaveta Tabaksblat:** Investigation; writing – review and editing. **Lene Weber Vestermark:** Investigation; writing – review and editing. **Johanna Svensson:** Investigation; writing – review and editing. **Ulrich Knigge:** Investigation; writing – review and editing. **Luís Nunes:** Investigation; writing – review and editing. **Bengt Glimelius:** Investigation; writing – review and editing. **Per Pfeiffer:** Investigation; writing – review and editing. **Kristine Aasebø:** Investigation; writing – review and editing. **Jörg Assmus:** Writing – review and editing. **Erik Vassella:** Investigation; writing – review and editing. **Inger Marie Bowitz Lothe:** Investigation; writing – review and editing. **Anne Couvelard:** Investigation; writing – review and editing. **Aurel Perren:** Investigation; writing – review and editing. **Stian Knappskog:** Writing – original draft; supervision. **Halfdan Sorbye:** Supervision; conceptualization; investigation; writing – original draft; data curation.

## FUNDING INFORMATION

The Western Norway Regional Health Authority, Nordic Cancer Union, Novartis, Ipsen, and the Norwegian Cancer Society supported this work.

## CONFLICT OF INTEREST STATEMENT

SM, SWL, GOH, AS, EH, ET, LWV, JS, UK, LN, BG, PP, KA, JA, EV, IMBL, AC, AP: No disclosures. ML: Unrestricted research funding by Scandion Oncology A/S, Copenhagen, Denmark; international advisory board member, Alivia AB, Stockholm, Sweden. SK: Received research funding for other projects from AstraZeneca, Pfizer, and Illumina, and lecture honoraria from AstraZeneca, Pfizer, Pierre Fabre, and Novartis. HS: Advisory board for Novartis, Ipsen, and BMS, and speakers' honoraria from Pierre Fabre, Ipsen, SAM Nordic, and Daiichi Sankyo.

## ETHICS STATEMENT

The NORDIC NEC study was approved by the ethics committees in Denmark (Region Hovedstaden H‐4‐2012‐108), Sweden (REC Uppsala Dnr 2012/285), and Norway (REK Vest 2012/940). The SPCRC study was approved by the ethics committees in Denmark (The Regional Scientific Ethical Committees for Southern Denmark S‐20162000‐48), Sweden (Regional Ethical Committee Uppsala, Ups 03‐303), and Norway (REK VEST 2009/2052). Both studies were conducted in accordance with the Declaration of Helsinki. Written informed consent was obtained from all patients.

## Supporting information


**Table S1.** Co‐occurring *BRAF* and *KRAS* mutations in metastatic colorectal adenocarcinoma patients.
**Table S2.** Annotation of colorectal driver mutations in the metastatic colorectal neuroendocrine carcinoma (CR‐NEC) cohort.
**Table S3.** Annotation of colorectal driver mutations in the metastatic colorectal adenocarcinoma (CR‐AC) cohort.
**Table S4.** Regions covered by the 46 gene hotspot panel applied in the metastatic colorectal adenocarcinoma cohort.
**Table S6.** Selected baseline characteristics in 163 metastatic colorectal neuroendocrine carcinoma patients receiving first‐line palliative chemotherapy according to primary site.
**Table S7.** Cox regression analysis on baseline characteristics for median progression‐free survival (A) and median overall survival (B) in metastatic colorectal neuroendocrine carcinoma receiving first‐line chemotherapy.
**Table S8.** Response rate and survival outcomes in metastatic colorectal neuroendocrine carcinoma patients receiving second, third, and fourth‐line palliative chemotherapy.
**Table S9.** Treatment outcomes for metastatic colorectal neuroendocrine carcinoma receiving first‐line palliative chemotherapy according to mutation status.
**Table S10.** Molecular alterations stratified by primary site in metastatic colorectal neuroendocrine carcinoma (CR‐NEC) and metastatic colorectal adenocarcinoma (CR‐AC).
**Figure S1.** Patient selection for the metastatic colorectal neuroendocrine carcinoma (CR‐NEC) and the metastatic colorectal adenocarcinoma (CR‐AC) cohort.
**Figure S2A.** PFS in CR‐NEC receiving first‐line chemotherapy according to primary site.
**Figure S2B.** OS in CR‐NEC receiving first‐line chemotherapy according to primary site.
**Figure S3.** PFS in CR‐NEC receiving first‐line chemotherapy according to performance status.
**Figure S4.** Final multivariable model for survival following first‐line chemotherapy in CR‐NEC.

**Table S5.** Uploaded as separate excel file.

## Data Availability

The data that support this study are available from the corresponding author upon reasonable request.
