# Supplementary material for: Molecular–clinical characteristics and treatment outcomes in 163 metastatic colorectal neuroendocrine carcinomas with a comparison to colorectal adenocarcinomas
Source: Int J Cancer. 2026 Feb 5;158(12):3217–31. doi: 10.1002/ijc.70367 (PMC13106932; doi:10.1002/ijc.70367)
Supplement: Supplementary file 1 — Table S1. Co‐occurring BRAF and KRAS mutations in metastatic colorectal adenocarcinoma patients. Table S2. Annotation of colorectal driver mutations in the metastatic colorectal neuroendocrine carcinoma (CR‐NEC) cohort. Table S3. Annotation of colorectal driver mutations in the metastatic colorectal adenocarcinoma (CR‐AC) cohort. Table S4. Regions covered by the 46 gene hotspot panel applied in the metastatic colorectal adenocarcinoma cohort. Table S6. Selected baseline characteristics in 163 metastatic colorectal neuroendocrine carcinoma patients receiving first‐line palliative chemotherapy according to primary site. Table S7. Cox regression analysis on baseline characteristics for median progression‐free survival (A) and median overall survival (B) in metastatic colorectal neuroendocrine carcinoma receiving first‐line chemotherapy. Table S8. Response rate and survival outcomes in metastatic colorectal neuroendocrine carcinoma patients receiving second, third, and fourth‐line palliative chemotherapy. Table S9. Treatment outcomes for metastatic colorectal neuroendocrine carcinoma receiving first‐line palliative chemotherapy according to mutation status. Table S10. Molecular alterations stratified by primary site in metastatic colorectal neuroendocrine carcinoma (CR‐NEC) and metastatic colorectal adenocarcinoma (CR‐AC). Figure S1. Patient selection for the metastatic colorectal neuroendocrine carcinoma (CR‐NEC) and the metastatic colorectal adenocarcinoma (CR‐AC) cohort. Figure S2A. PFS in CR‐NEC receiving first‐line chemotherapy according to primary site. Figure S2B. OS in CR‐NEC receiving first‐line chemotherapy according to primary site. Figure S3. PFS in CR‐NEC receiving first‐line chemotherapy according to performance status. Figure S4. Final multivariable model for survival following first‐line chemotherapy in CR‐NEC. [file IJC-158-3217-s001.docx]

**Molecular-Clinical Characteristics and Treatment Outcomes in 163 Metastatic Colorectal Neuroendocrine Carcinomas with a Comparison to Colorectal Adenocarcinomas**

Siren Morken, Seppo W. Langer, Geir Olav Hjortland, Anna Sundlöv, Eva Hofsli, Morten Ladekarl, Elizaveta Tabaksblat, Lene Weber Vestermark, Johanna Svensson, Ulrich Knigge, Luís Nunes, Bengt Glimelius, Per Pfeiffer, Kristine Aasebø, Jörg Assmus, Erik Vassella, Inger Marie Bowitz Lothe, Anne Couvelard, Aurel Perren, Stian Knappskog and Halfdan Sorbye.

**Table of content**

[Material & methods 2](#_Toc219901853)

[Molecular data in the metastatic colorectal NEC cohort 2](#_Toc219901854)

[Molecular data in the metastatic colorectal AC cohort 2](#_Toc219901855)

[Annotation of driver events in the metastatic colorectal NEC and colorectal AC cohort 3](#_Toc219901856)

[Comparison of molecular data in the metastatic colorectal NEC and colorectal AC cohort 3](#_Toc219901857)

[Supplementary tables 5](#_Toc219901858)

[**Table S1** 5](#_Toc219901859)

[**Table S2** 6](#_Toc219901860)

[**Table S3** 7](#_Toc219901861)

[**Table S4** 8](#_Toc219901862)

[**Table S5** Uploaded as separate excel file 9](#_Toc219901863)

[**Table S6** 10](#_Toc219901864)

[**Table S7** 11](#_Toc219901865)

[**Table S8** 12](#_Toc219901866)

[**Table S9** 13](#_Toc219901867)

[**Table S10** 14](#_Toc219901868)

[Supplementary figures 15](#_Toc219901869)

[**Figure S1** 15](#_Toc219901870)

[**Figure S2A** 15](#_Toc219901871)

[**Figure S2B** 16](#_Toc219901872)

[**Figure S3** 16](#_Toc219901873)

[**Figure S4** 17](#_Toc219901874)

[References 18](#_Toc219901875)

# **Material & methods**

## *Molecular data in the metastatic colorectal NEC cohort*

Molecular data for the colorectal NEC (CR-NEC) cohort was collected by using three approaches:

1. For 78 patients, BRAF, *K*RAS, APC, TP53, and RB1 mutation status was available through our previous targeted panel NGS-based (next generation sequencing) analysis^1^, applying a custom-made 360 cancer gene panel, previously described^2^ and run in house on the Illumina MiSeq platform. In this analysis, the entire coding regions of all genes were covered. MSI status was available for 61 patients through our previous MSI analysis with the Promega MSI Analysis System (Version 1.2, Promega). *RB1* copy number (deletion) status was assessed using FACETS for 58 patients, previously described^1^.
2. BRAF (n=45), KRAS (n=49), and MSI (n=48) status were available from case report forms, as patients had undergone routine molecular diagnostics at their local study centre.
3. For patients lacking available tumour tissue, NGS-based analysis was performed on liquid biopsies (plasma) applying the Illumina TSO500 ctDNA panel making mutation and MSI status available for another 23 patients. Sequencing libraries were prepared from >30 ng plasma cfDNA, using the TSO500 ctDNA v1 kit (Illumina), using UMIs for indexing, enabling assessment of depth in unique molecules. Sequencing was performed using a NovaSeq6000 instrument (Illumina), in 32-plex of samples on S4 flow cells, aiming for a minimum coverage of 1300x. Mapping, mutation calling, and preliminary annotation were performed using the Dragen-server’s (Illumina) built-in TSO500 pipeline. Post-processing, filters were applied in-house to remove benign variants and polymorphisms. These included removal of variants with >0.1% minor allele frequency (MAF) in the gnomAD database, and intronic and intergenic variants. Further, all retained variants were required to have a minimum of 3 reads (absolute count), equivalent to a variant allele frequency (VAF) of 0.25% in data with 1300x coverage. Mutations likely to be linked to clonal haematopoiesis of indeterminate potential (CHIPs), were removed by manual curation. This included variants in *DNMT3A*, *TET2* and *ASXL1* genes. The remaining mutations were classified into tiers, largely based on the levels in the OncoKB database: mutations listed as level 1 or 2 in OncoKB were considered drivers. In addition, some canonical driver mutations that are not targetable, and therefore not listed as level 1 or 2 in OncoKB, were included by manual curation.

For a subset of cases, molecular data was available from more than one method (*BRAF* n=24, *KRAS* n=28, MSI status n=25). In cases of discordance between methods, one or more positive results were considered sufficient to classify as a positive result for the patient’s status. In total, *KRAS* and *BRAF* status was available for 122 patients, while *APC*, TP53, and RB1 status was available for 101 patients.

## *Molecular data in the metastatic colorectal AC cohort*

Molecular data for the colorectal adenocarcinoma (CR-AC) cohort was collected using three approaches:

1. *BRAF* (n=259) and *KRAS* (n=257) mutational status were assessed by pyrosequencing, using polymerase chain reaction (PCR) primers specially designed to generate reads covering *BRAF* codon 600 and *KRAS* codon 12/13, as previously described^3^. MSI status was available for 262 patients and determined via a combination of immunohistochemistry (IHC) and PCR technique using MSI Analysis System (Version 1.2, Promega), as previously described^4,5^.
2. *BRAF* IHC was performed for 262 patients, as previously described^5^.
3. For 234 patients, *BRAF*, *KRAS*, *APC*, *TP53*, and *RB1* status were assessed through a custom-designed Ampliseq hotspot panel (Thermo-Fischer Scientific, Waltham, MA, USA) covering mutations hot spots in 46 cancer related genes, as previously described^4^.

Regarding *BRAF* mutations, four cases had only IHC status avaiable; for the rest more than one method was avaiable. Regarding *KRAS* mutations, 22 patients only had pyrosequencing status avaiable, while the rest also had NGS status avaiable. In discordant cases, any positive *BRAF* or *KRAS* status was considered valid, regardless of the method.

Seven CR-AC patients had a co-occurring *BRAF* and *KRAS* mutation (**Supplementary Table S7**). Patients 1103 and 2071 had a *KRAS* mutation co-occurring with a *BRAF* non-V600E mutation, which we have defined as driver mutations in this cohort. Patients 3131 and 3164 had a *KRAS* mutation and only had positive *BRAF* status on immunohistochemistry (BRAF_IHC). Despite the discordance between the different methods applied, we have chosen to regard these two patients as *BRAF* mutated, even if we acknowledge the potential limitation of relying on IHC alone.

## *Annotation of driver events in the metastatic colorectal NEC and colorectal AC cohort*

For these two cohorts we selected five genes known to be canonical driver genes in colorectal cancer. Each mutation within these genes was annotated as a likely driver mutation, contributing to colorectal cancer oncogenesis based on the criteria described below. Details on the annotation of driver mutations can be found in **Supplementary Table S8** for the CR-NEC cohort and in **Supplementary Table S9** for the CR-AC cohort.

Oncogenes *BRAF* and *KRAS*:

Canonical oncogenic mutations in recurrent hotspots: *BRAF* V600E, *KRAS* codons 12 and 13. Alongside, recurrent somatic mutations previously confirmed at this locus in cosmic with a mutation count of ≥ 10 for *BRAF* and ≥ 10 for *KRAS*.

Tumour suppressor genes *APC*, *TP53* and *RB1*:

Likely damaging events in tumour suppressor genes: Truncating (nonsense and frameshift) mutations were considered driver events, along with somatic mutations previously confirmed at this locus in COSMIC with a mutation count of ≥ 10 for *APC* and ≥ 5 for *RB1*. All missense mutations in *TP53* were considered driver events. We had no somatic *TP53* missense mutations in codons 72 (which is a known polymorphic germline site). Splice-site mutations were considered a driver event when detected around 3 base pairs from a splice site (two observed cases for *TP53*). Silent mutations (synonymous) were not considered driver events.

## *Comparison of molecular data in the metastatic colorectal NEC and colorectal AC cohort*

*BRAF* and *KRAS* mutations were assessed using multiple methods in the two cohorts, and we consider that the majority of *BRAF* and *KRAS* mutations are covered in both groups. As most of these mutations are associated with well-known hot spots, *BRAF* V600E and *KRAS* codons 12 and 13, there was a complete overlap between the *BRAF*/*KRAS* mutations identified in the CR-NEC cohort and the coverage of the hotspot panel applied for the CR-AC cohort.

The comparison of *APC*, *TP53*, and *RB1* status between the two cohorts was restricted to the coverage of the 46 gene hotspot panel applied in the CR-AC cohort. The hotspot panel did not cover 17/49 *APC* mutations, 4/ 64 *TP53* mutations, and 7/15 *RB1* mutations found by whole-exon NGS in the CR-NEC cohort. To enable a valid comparison of *APC, TP53*, and *RB1* mutation status between the two cohorts, only mutations covered by the hotspot panel were considered. CR-NEC cases with mutations not covered by the hotspot panel were considered wild-type for the sole purpose of comparing mutation frequency in the two cohorts. Regions covered, within these 5 genes, by the hot spot panel can be found in **Supplementary Table S10**.

# **Supplementary tables**

**Table S1** Co-occurring *BRAF* and *KRAS* mutations in metastatic colorectal adenocarcinoma patients

**Table S2** Annotation of colorectal driver mutations in the metastatic colorectal neuroendocrine carcinoma (CR-NEC) cohort

| **CR-NEC cohort** | **N** | **Driver** | **Comment** |
| --- | --- | --- | --- |
| ***BRAF mutations*** |  |  |  |
| Missense: p.V600E | 32 | yes | Known hotspot |
| Deletion-Insertion: p.Phe242_Ala246delinsLeu | 1 | no | Likely loss-of-function or no functional impact |
| Driver *BRAF* mutations | 32 |  |  |
| ***KRAS* mutations** |  |  |  |
| Missense: codon 12 and 13 | 24 | yes | Known hotspot |
| Missense: | 10 |  |  |
| p.A59T | 1 | yes | Cosmic count 47 |
| p.Q61H | 2 | yes | Known hotspot |
| p.Q61R | 2 | yes | Known hotspot |
| p.K117N | 2 | yes | Cosmic count 64 |
| p.A146T | 3 | yes | Known hotspot |
| Deletion-Insertion: p.A11delinsGA | 1 | yes | Recurrence of indels in locus |
| Missing | 7 | yes | Data from case report forms: type of KRAS mutation not specified |
| Driver *KRAS* mutations | 42 |  |  |
| ***APC* mutations** |  |  |  |
| Nonsense or frameshift | 47 | Yes |  |
| Germline | 2 | Yes |  |
| Missense: p.S1042G | 1 | No | Cosmic count 0 |
| APC driver mutations | 49 |  |  |
| ***TP53* mutations** |  |  |  |
| Nonsense or frameshift | 10 | yes |  |
| Missense: | 52 |  | All *TP53* missense mutations were considered driver mutations. |
| Splice-site | 1 | yes | Likely loss-of-function |
| Deletion: p.191_192del | 1 | yes | Likely loss-of-function |
| Driver *TP53* mutations | 64 |  |  |
| ***RB1* mutations** |  |  |  |
| Nonsense or frameshift | 13 | yes |  |
| Splice-site mutation | 1 | yes | Likely loss-of-function |
| Missense: p.V439I | 1 | no | Cosmic count 0 |
| In frame deletion: p.704_704del | 1 | no |  |
| *RB1* driver mutations | 14 |  |  |

**Table S3** Annotation of colorectal driver mutations in the metastatic colorectal adenocarcinoma (CR-AC) cohort

| **CR-AC cohort** | **N** | **Driver** | **Comment** |
| --- | --- | --- | --- |
| ***BRAF mutations*** |  |  |  |
| Missense: |  |  |  |
| p.V600E | 49 | yes | Known hotspot |
| p.G466E | 2 | yes | Cosmic count 45 |
| p.D594N | 1 | yes | Cosmic count 87 |
| Driver *BRAF* mutations | 52 |  |  |
| ***KRAS* mutations** |  |  |  |
| Missense codon 12 or 13 | 107 | yes | Known hotspot |
| Missense codon 12 or 13 + other missense | 2 | yes | Known hotspot |
| Missense: |  |  |  |
| p.V14I | 1 | yes | Cosmic count 39 |
| p.G15S | 1 | yes | Cosmic count 4 (multiple drivers in the same codon and close by codons 12 and 13) |
| p.A59T | 1 | yes | Cosmic count 47 |
| p.Q61H | 1 | yes | Known hotspot |
| p.Q61R | 1 | yes | Known hotspot |
| p.A146P | 1 | yes | Known hotspot |
| p.A146T | 1 | yes | Known hotspot |
| p.A146V | 1 | yes | Known hotspot |
| Driver *KRAS* mutations | 117 |  |  |
| ***APC* mutations** |  |  |  |
| Nonsense and/or frameshift | 78 | yes |  |
| Nonsense or frameshift + missense | 4 | yes |  |
| APC driver mutations | 82 |  |  |
| ***TP53* mutations** |  |  |  |
| Nonsense or frameshift | 20 | yes |  |
| Nonsense or frameshift + missense | 4 | yes |  |
| Missense | 102 | yes | All *TP53* missense mutations were considered driver mutations |
| Driver *TP53* mutations | 126 |  |  |
| ***RB1* mutations** |  |  |  |
| Nonsense or frameshift | 2 | yes |  |
| Nonsense + missense | 1 | yes |  |
| Missense: |  |  |  |
| p.E322K | 2 | yes | Cosmic count 9 |
| p.V456I | 1 | no | Cosmic count 1 |
| p.R661Q | 1 | no | Cosmic count 4 |
| p.I680T | 1 | yes | Cosmic count 11 |
| *RB1* driver mutations | 6 |  |  |

**Table S4** Regions covered by the 46 gene hotspot panel applied in the metastatic colorectal adenocarcinoma cohort

| Amplicon ID | Genome | Forward Primer | Reverse Primer | Gene | Chr. | Insert Start | Insert Stop |
| --- | --- | --- | --- | --- | --- | --- | --- |
| APC_1 | hg19 | GAGAGAACGCGGAATTGGTCTA | GTATGAATGGCTGACACTTCTTCCA | *APC* | chr5 | 112173872 | 112173962 |
| APC_2 | hg19 | AGCACTGATGATAAACACCTCAAGTT | ATCTTCTTGACACAAAGACTGGCT | *APC* | chr5 | 112174558 | 112174666 |
| APC_3 | hg19 | TTCATTATCATCTTTGTCATCAGCTGAA | TTTGGTTCTAGGGTGCTGTGAC | *APC* | chr5 | 112175144 | 112175268 |
| APC_4 | hg19 | GCAGACTGCAGGGTTCTAGTT | GTGAACTGACAGAAGTACATCTGCT | *APC* | chr5 | 112175316 | 112175443 |
| APC_5 | hg19 | AGCCCCAGTGATCTTCCAGATA | CCCTCTGAACTGCAGCATTTACT | *APC* | chr5 | 112175568 | 112175703 |
| APC_6 | hg19 | AGAGGGTCCAGGTTCTTCCA | TCATTTTCCTGAACTGGAGGCATT | *APC* | chr5 | 112175741 | 112175862 |
| APC_7 | hg19 | ATGAAACAGAATCAGAGCAGCCTAAA | CGTGATGACTTTGTTGGCATGG | *APC* | chr5 | 112175921 | 112176035 |
| BRAF_1 | hg19 | CATACTTACCATGCCACTTTCCCTT | TTTCTTTTTCTGTTTGGCTTGACTTGA | *BRAF* | chr7 | 140481392 | 140481515 |
| BRAF_2 | hg19 | CCACAAAATGGATCCAGACAACTGT | GCTTGCTCTGATAGGAAAATGAGATCTA | *BRAF* | chr7 | 140453103 | 140453221 |
| KRAS_1 | hg19 | CAAAGAATGGTCCTGCACCAGTAATAT | AGGCCTGCTGAAAATGACTGAATATAA | *KRAS* | chr12 | 25398187 | 25398304 |
| KRAS_2 | hg19 | TCCTCATGTACTGGTCCCTCATT | GTAAAAGGTGCACTGTAATAATCCAGACT | *KRAS* | chr12 | 25380261 | 25380364 |
| KRAS_3 | hg19 | CAGATCTGTATTTATTTCAGTGTTACTTACCT | GACTCTGAAGATGTACCTATGGTCCTA | *KRAS* | chr12 | 25378550 | 25378658 |
| RB1_1 | hg19 | ACTTTTTTCTATTCTTTCCTTTGTAGTGTCCATA | CCTTTCCAATTTGCTGAAGAGTGC | *RB1* | chr13 | 48919224 | 48919312 |
| RB1_2 | hg19 | GCATTGGTGCTAAAAGTTTCTTGGAT | AAGCAGAGAATGAGGGAGGAGTA | *RB1* | chr13 | 48923140 | 48923255 |
| RB1_3 | hg19 | GCTGAGAGATGTAATGACATGTAAAGGA | CCATGTGCAATACCTGTCTATAGAATCA | *RB1* | chr13 | 48941602 | 48941724 |
| RB1_4 | hg19 | TGAGACAACAGAAGCATTATACTGCTTT | CTGGAGTGTGTGGAGGAATTACATT | *RB1* | chr13 | 48942598 | 48942711 |
| RB1_5 | hg19 | CGATACAAACTTGGAGTTCGCTTG | CTTGATGCCTTGACCTCCTGAT | *RB1* | chr13 | 48953754 | 48953874 |
| RB1_6 | hg19 | AGAAGGCAACTTGACAAGAGAAATGATA | CAATAATTTGTTAGCCATATGCACATGAATGA | *RB1* | chr13 | 48955526 | 48955605 |
| RB1_7 | hg19 | CTGGGAAAATTATGCTTACTAATGTGGTTT | ACAAGCAGATTCAAGGTGATCAGTT | *RB1* | chr13 | 49027106 | 49027178 |
| RB1_8 | hg19 | AGTAAAAATGACTAATTTTTCTTATTCCCACAGTGTA | TGCCTGTCTCTCATGAGTTCATACT | *RB1* | chr13 | 49033828 | 49033934 |
| RB1_9 | hg19 | AACAAAACCATGTAATAAAATTCTGACTACTTT | GAGGAAGATCCTTGTATGCTGTTAC | *RB1* | chr13 | 49037847 | 49037932 |
| RB1_10 | hg19 | TCTTCCTCAGACATTCAAACGTGTTT | ACCTACCCTGGTGGAAGCATA | *RB1* | chr13 | 49039150 | 49039232 |
| TP53_1 | hg19 | TCCACTCACAGTTTCCATAGGTCT | GTTGGAAGTGTCTCATGCTGGAT | *TP53* | chr17 | 7579854 | 7579960 |
| TP53_2 | hg19 | GGCTGTCCCAGAATGCAAGAA | GATGAAGCTCCCAGAATGCCA | *TP53* | chr17 | 7579351 | 7579485 |
| TP53_3 | hg19 | TGCACAGGGCAGGTCTTG | CCGTCTTCCAGTTGCTTTATCTGT | *TP53* | chr17 | 7578517 | 7578601 |
| TP53_4 | hg19 | ACCAGCCCTGTCGTCTCT | GTGCAGCTGTGGGTTGATTC | *TP53* | chr17 | 7578353 | 7578483 |
| TP53_5 | hg19 | CCAGTTGCAAACCAGACCTCA | AGGCCTCTGATTCCTCACTGAT | *TP53* | chr17 | 7578181 | 7578298 |
| TP53_6 | hg19 | GGCTCCTGACCTGGAGTCTT | CTCATCTTGGGCCTGTGTTATCTC | *TP53* | chr17 | 7577509 | 7577612 |
| TP53_7 | hg19 | CGCTTCTTGTCCTGCTTGCT | TTCTCTTTTCCTATCCTGAGTAGTGGT | *TP53* | chr17 | 7577016 | 7577151 |
| TP53_8 | hg19 | GGAAGGGGCTGAGGTCACT | CCCCTCCTCTGTTGCTGC | *TP53* | chr17 | 7573924 | 7574035 |

**Table S5** Uploaded as separate excel file

**Table S6** Selected baseline characteristics in 163 metastatic colorectal neuroendocrine carcinoma patients receiving first-line palliative chemotherapy according to primary site

|  |  | **All patients N = 163** | **Colon right N = 62** | **Colon left N = 22** | **Rectum N = 79** |
| --- | --- | --- | --- | --- | --- |
|  | Valid cases | N (%) | N (%) | N (%) | N (%) |
| Age, median (range) | 163 | 67.7 (29.6-87.7) | 69.1 (41.8-87.7) | 66.5 (29.6-82.3) | 65.9 (30.9-83.5) |
| Male | 163 | 94 (58) | 29 (47) | 12 (54) | 53 (67) |
| Prior other cancer ^a^ | 163 | 32 (20) | 8 (13) | 6 (27) | 18 (23) |
| Smoker-Prior smoker ^b^ | 143 | 73 (51) | 30 (56) | 9 (45) | 34 (49) |
| Performance status ^b^ | 159 |  |  |  |  |
| 0 |  | 50 (31) | 22 (36) | 8 (38) | 20 (26) |
| 1 |  | 73 (46) | 27 (44) | 7 (34) | 39 (51) |
| 2 |  | 25 (16) | 9 (15) | 3 (14) | 13 (17) |
| 3 |  | 11 (7) | 3 (5) | 3 (14) | 5 (6) |
| Ki-67, median (range) | 163 | 90 (23-100) | 90 (30-100) | 90 (50-100) | 90 (23-100) |
| ≤ 55% |  | 13 (8) | 4 (6) | 1 (4) | 8 (10) |
| > 55% |  | 150 (92) | 58 (94) | 21 (96) | 71 (90) |
| Cell type^b^ | 155 |  |  |  |  |
| Large cell |  | 99 (64) | 47 (77) | 15 (75) | 37 (50) |
| Small cell |  | 56 (36) | 14 (23) | 5 (25) | 37 (50) |
| Primary tumor resected | 163 | 58 (36) | 26 (42) | 11 (50) | 21 (27) |
| Synchronous metastasis | 163 | 140 (86) | 53 (85) | 20 (91) | 67 (85) |
| Sites of metastasis | 163 |  |  |  |  |
| Liver |  | 129 (79) | 47 (76) | 19 (86) | 63 (80) |
| Lymph nodes |  | 61 (37) | 21 (34) | 7 (32) | 33 (42) |
| Lung |  | 29 (18) | 11 (18) | 3 (14) | 15 (19) |
| Bone |  | 24 (15) | 4 (6) | 3 (14) | 17 (21) |
| Peritoneum |  | 6 (4) | 5 (8) | 0 | 1 (1) |
| Other ^c^ |  | 14 (9) | 9 (14) | 0 | 5 (6) |
| Development of brain metastasis ^b^ | 155 | 15 (10) | 2 (3) | 2 (10) | 11 (14) |
| FDG-PET uptake ^b^ | 58 | 58 (100) | 19 (100) | 8 (100) | 31 (100) |
| SRI + Octreoscan/Ga-PET > liver ^b,d^ | 36 | 7 (19) | 1 (8) | 2 (29) | 4 (23) |
| CgA serum > UNL ^b^ | 122 | 58 (47) | 23 (43) | 6 (54) | 29 (51) |
| NSE > UNL ^b^ | 82 | 59 (72) | 26 (70) | 6 (86) | 27 (71) |
| LDH > UNL ^b^ | 147 | 69 (47) | 24 (42) | 11 (58) | 34 (48) |
| ALP > UNL ^b^ | 158 | 89 (56) | 33 (55) | 12 (57) | 44 (57) |
| Platelets > 400 x 10 9/L ^b^ | 160 | 42 (26) | 19 (31) | 8 (38) | 15 (19) |
| WBC >10 x 10 9/L ^b^ | 160 | 53 (33) | 21 (35) | 8 (38) | 24 (30) |
| CRP ≥ 10 ^b^ | 135 | 82 (61) | 28 (54) | 14 (82) | 40 (61) |

Abbreviations: ALP, alkaline phosphatase; CgA, chromogranin A; CRP, C-reactive protein; FDG-PET, fluorodeoxyglucose-positron emission tomography; Ga-PET, Gallium-positron emission tomography; LDH, lactate dehydrogenase; NSE, neuron specific enolase; SRI, somatostatin receptor imaging; UNL, upper normal limit; WBC, white blood cells.

^a^Colon/rectum n = 13, breast n = 5, prostate n = 4, basal cell carcinoma n = 2, bladder n =2, skin n = 1, esophagus n =1, uterine/ovarian n =1, testis n = 1, renal pelvis n = 1, mantle cell lymphoma n = 1, vulva n = 1, anorectal NET n = 1 (one patients had both prostate and sigmoid cancer).

^b^Percentage as a fraction of examined patients.

^c^Adrenal n = 3, ovarium n = 3, pancreas n = 2, kidney n = 2, adipose tissue n = 2, small intestine n = 1, pelvis n = 1, prostate n = 1, muscle n = 1 (two patients were registered with two other metastatic locations).

^d^68Ga-DOTATATE PET/CT (n = 17), 111In-octreotide SPECT (n = 26), both imaging modalities (n = 7, with same result).

**Table S7** Cox regression analysis on baseline characteristics for median progression-free survival (A) and median overall survival (B) in metastatic colorectal neuroendocrine carcinoma receiving first-line chemotherapy

**(A)**

**(B)**

**Table S8** Response rate and survival outcomes in metastatic colorectal neuroendocrine carcinoma patients receiving second, third and fourth-line palliative chemotherapy

|  | **Second-line N = 90** | | **Third-line N = 42** | | **Fourth-line N = 11** | |
| --- | --- | --- | --- | --- | --- | --- |
|  | Valid cases | N (%) | Valid cases | N (%) | Valid cases | N (%) |
| Performance status | 80 |  |  |  |  |  |
| 0 |  | 14 (17) |  | - |  | - |
| 1 |  | 43 (54) |  | - |  | - |
| 2 |  | 22 (28) |  | - |  | - |
| 3 |  | 1 (1) |  | - |  | - |
| Regime | 90 |  | 42 |  | 11 |  |
| Platinum/etoposide |  | 8 (9) |  | 3 (7) |  | 0 |
| CAPTEM |  | 30 (33) |  | 1 (2) |  | 2 (18) |
| Other |  | 52 (58) ^b^ |  | 38 (90) ^c^ |  | 9 (81) ^d^ |
| Best response | 90 |  | 42 |  | 11 |  |
| Response (CR+PR) |  | 13 (14) |  | 4 (9) |  | 0 |
| SD |  | 11 (12) |  | 11 (27) |  | 2 (18) |
| PD^a^ |  | 61 (68) |  | 23 (55) |  | 8 (73) |
| NE/NA |  | 5 (6) |  | 4 (9) |  | 1 (9) |
| Median duration of response, months | 8 | 3.5 (2-9) | 2 | 1.5 (1-2) |  | - |
| Median PFS (95% CI), months | 90 | 2.04 (1.81-2.53) | 42 | 2.02 (1.81-3.29) | 11 | 1.97 (1.81-2.92) |
| Median OS (95% CI), months | 90 | 4.57 (3.57-7.39) | 42 | 4.88 (3.31-6.44) | 11 | 5.09 (2.92-11.6) |

Abbreviations: CAPTEM, capecitabine/temozolomide; CR, complete response; NE/NA, not evaluated/not assessed; OS, overall survival; PD, progressive disease; PR, partial response; SD, stable disease. PFS, progression free survival.

^a^Radiologic (RECIST) and clinical progressive disease.

^b^FOLFIRI n = 10, topotecan n = 10, temozolomide n = 7, FLIRI n = 6, FOLFIRINOX n = 5, capecitabine n = 4, FOLFOX n = 3, Oxaliplatin/5-FU n = 2, Irinotecan/capecitabine n = 1, paclitaxel n = 1, vemurafenib/panitumumab n = 1, ACO n = 1, everolimus n = 1.

^c^Temozolomide n = 6, FLIRI n = 5, FOLFIRI n = 4, FLOX n = 3, docetaxel n = 3, FOLFIRINOX n = 2, capecitabine n = 2, ACO n = 2, CAV n = 2, etoposide n = 1, FOLFIRI/cetuximab n = 1, atezolizumab n = 1, irinotecan n = 1, IRIS n = 1, lonsurf n = 1, paclitaxel n = 1, topotecan n = 1, gamma knife n = 1.

^d^Capecitabine n = 2, topotecan n = 2, FLOX n = 1, FOLFOX n = 1, etoposide n = 1, temozolomide n = 1, paclitaxel n = 1.

**Table S9** Treatment outcomes for metastatic colorectal neuroendocrine carcinoma receiving first-line palliative chemotherapy according to mutation status

|  |  | **CR + PR (%)** | **SD (%)** | **PD (%)** | **NA/NE (%)** | **Median PFS (95% CI), months** | **Median OS (95% CI), months** |
| --- | --- | --- | --- | --- | --- | --- | --- |
|  | Valid cases |  |  |  |  |  |  |
| ***BRAF*** | 122 |  |  |  |  |  |  |
| Wild type | 90 | 28 (31) | 12 (13) | 43 (48) | 7 (8) | 2.7 (2.2-4.1) | 8.7 (6.2-10.7) |
| Colon right | 21 | 4 (19) | 4 (19) | 13 (62) | - | 2.6 (2.1-5.8) | 8.6 (6.7-12.3) |
| Colon left | 11 | 4 (36) | 1 (9) | 6 (55) | - | 2.1 (0.9-5.7) | 5.7 (2.1-12.2) |
| Rectum | 58 | 20 (35) | 7 (12) | 24 (41) | 7 (12) | 3.3 (2.0-4.5) | 8.9 (5.7-13.0) |
| Mutated | 32 | 4 (12) | 10 (31) | 12 (38) | 6 (19) | 2.3 (1.7-5.2) | 4.8 (2.7-9.9) |
| Colon right | 25 | 4 (16.0) | 7 (28.0) | 9 (36.0) | 5 (20.0) | 2.4 (1.8-5.8) | 5.6 (2.7-12.0) |
| Colon left | 6 | - | 3 (50) | 2 (33) | 1 (17) | 2.5 (1.3-5.5) | 3.1 (1.6-10.2) |
| Rectum | 1 | - | - | 1 (100) | - | 1.4 (NA-NA) | 2.3 (NA-NA) |
| ***KRAS*** | 122 |  |  |  |  |  |  |
| Wild type | 80 | 22 (27) | 16 (20) | 34 (43) | 8 (10) | 2.8 (2.1-4.2) | 7.7 (5.6-10.7) |
| Colon right | 31 | 4 (13) | 8 (26) | 14 (45) | 5 (16) | 2.4 (1.9-5.2) | 6.4 (3.9-12.0) |
| Colon left | 13 | 2 (15) | 3 (23) | 7 (54) | 1 (8) | 1.8 (1.3-5.5) | 3.4 (1.8-10.2) |
| Rectum | 36 | 16 (44) | 5 (14) | 13 (36) | 2 (6) | 3.8 (2.5-6.0) | 11.2 (6.9-16.2) |
| Mutated | 42 | 10 (24) | 6 (14) | 21 (50) | 5 (12) | 2.4 (1.9-4.1) | 7.1 (5.7-10.0) |
| Colon right | 15 | 4 (27) | 3 (20) | 8 (53) | - | 2.7 (2.0-6.8) | 8.0 (6.7-14.4) |
| Colon left | 4 | 2 (50) | 1 (25) | 1 (25) | - | 5.9 (0.7-NA) | 9.7 (3.3-NA) |
| Rectum | 23 | 4 (17) | 2 (9) | 12 (52) | 5 (22) | 1.9 (1.7-5.5) | 5.7 (1.8-10.3) |
| ***APC*** | 101 |  |  |  |  |  |  |
| Wild type | 52 | 11 (21) | 13 (25) | 21 (40) | 7 (14) | 2.6 (1.9-4.4) | 6.6 (3.7-12.3) |
| Colon right | 24 | 2 (8) | 9 (38) | 8 (33) | 5 (21) | 2.8 (2.1-5.8) | 7.1 (4.2-12.3) |
| Colon left | 6 | 1 (17) | 1 (17) | 3 (49) | 1 (17) | 1.6 (0.9-5.5) | 3.2 (2.1-15.2) |
| Rectum | 22 | 8 (36) | 3 (14) | 10 (46) | 1 (4) | 2.5 (1.7-5.7) | 6.5 (3.5-19.6) |
| Mutated | 49 | 14 (29) | 6 (12) | 24 (49) | 5 (10) | 2.4 (1.8-4.2) | 7.7 (5.7-10.3) |
| Colon right | 15 | 3 (20) | 2 (13) | 10 (67) | - | 2.1 (1.7-5.8) | 6.9 (5.7-11.9) |
| Colon left | 7 | 2 (29) | 2 (29) | 3 (42) | - | 4.1 (1.6-10.1) | 10.2 (1.6-12.2) |
| Rectum | 27 | 9 (33) | 2 (7) | 11 (41) | 5 (19) | 3.3 (1.8-5.5) | 8.8 (3.8-11.6) |
| ***TP53*** | 101 |  |  |  |  |  |  |
| Wild type | 37 | 10 (27) | 7 (19) | 18 (49) | 2 (5) | 2.6 (2.1-4.5) | 6.4 (3.8-11.9) |
| Colon right | 14 | 3 (21) | 2 (14) | 8 (58) | 1 (7) | 2.5 (2.1-6.8) | 6.7 (3.7-14.4) |
| Colon left | 4 | - | 2 (50) | 2 (50) | - | 3.7 (0.1-NA) | 7.9 (0.1-NA) |
| Rectum | 19 | 7 (37) | 3 (16) | 8 (42) | 1 (5) | 3.4 (2.0-5.8) | 6.2 (3.8-16.9) |
| Mutated | 64 | 15 (23) | 12 (19) | 27 (42) | 10 (16) | 2.3 (1.8-4.1) | 7.7 (5.6-10.2) |
| Colon right | 25 | 2 (8) | 9 (36) | 10 (40) | 4 (16) | 2.4 (1.7-5.8) | 7.4 (5.6-12.0) |
| Colon left | 9 | 3 (33) | 1 (11) | 4 (45) | 1 (11) | 2.1 (1.3-5.6) | 7.3 (2.1-11.1) |
| Rectum | 30 | 10 (33) | 2 (7) | 13 (43) | 5 (17) | 2.1 (1.7-6.4) | 8.9 (3.5-15.3) |
| ***RB1*** | 101 |  |  |  |  |  |  |
| Wild type | 87 | 23 (26) | 18 (21) | 37 (43) | 9 (10) | 2.4 (2.0-4.1) | 7.7 (5.7-10.2) |
| Colon right | 35 | 4 (11) | 11 (32) | 16 (46) | 4 (11) | 2.3 (1.8-3.9) | 7.4 (3.6-10.2) |
| Colon left | 11 | 3 (27) | 3 (27) | 4 (37) | 1 (9) | 4.1 (1.6-7.6) | 10.2 (2.8-15.2) |
| Rectum | 41 | 16 (39.0) | 4 (9.8) | 17 (41.5) | 4 (9.8) | 2.5 (1.7-4.5) | 8.2 (3.8-10.7) |
| Mutated | 14 | 2 (14) | 1 (7) | 8 (57) | 3 (22) | 2.9 (1.8-23.8) | 6.1 (2.1-23.9) |
| Colon right | 4 | 1 (25) | - | 2 (50) | 1 (25) | 3.2 (2.8-NA) | 6.9 (5.7-NA) |
| Colon left | 2 | - | - | 2 (100) | - | 0.5 (0.1-NA) | 1.1 (0.1-NA) |
| Rectum | 8 | 1 (12) | 1 (12) | 4 (50) | 2 (26) | 2.8 (0.7-7.0) | 6.1 (0.7-19.6) |
| ***RB1* deletion** | 58 |  |  |  |  |  |  |
| Wild type | 34 | 9 (26) | 7 (21) | 15 (44) | 3 (9) | 3.0 (2.4-5.8) | 8.4 (6.9-15.2) |
| Colon right | 19 | 3 (16) | 5 (26) | 8 (42) | 3 (16) | 2.8 (2.1-5.8) | 7.4 (5.7-11.9) |
| Colon left | 2 | - | 1 (50) | 1 (50) | - | 3.7 (1.8-NA) | 9.5 (3.7-NA) |
| Rectum | 13 | 6 (46) | 1 (8) | 6 (46) | - | 3.4 (1.9-7.7) | 10.5 (6.9-17.8) |
| Deletion | 24 | 8 (33) | 4 (17) | 10 (42) | 2 (8) | 2.0 (1.8-4.5) | 6.5 (2.7-11.1) |
| Colon right | 7 | 1 (14) | 2 (29) | 4 (57) | - | 1.9 (1.7-4.1) | 4.2 (2.7-10.6) |
| Colon left | 3 | 1 (33) | - | 2 (67) | - | 2.1 (0.9-NA) | 11.1 (2.1-NA) |
| Rectum | 14 | 6 (43) | 2 (14) | 4 (29) | 2 (14) | 2.7 (1.6-5.5) | 6.3 (1.7-13.0) |

Abbreviations: CI, confidence interval; CR, complete response; NE/NA, not evaluated/not assessed; OS, overall survival; PD, progressive disease; PFS, progression-free survival; PR, partial response; SD, stable disease.

**Table S10** Molecular alterations stratified by primary site in metastatic colorectal neuroendocrine carcinoma (CR-NEC) and metastatic colorectal adenocarcinoma (CR-AC)

|  | **Valid cases** | **All N (%)** | **Colon right N (%)** | **Colon left N (%)** | **Rectum N (%)** |
| --- | --- | --- | --- | --- | --- |
| ***BRAF* mutated** |  |  |  |  |  |
| CR-NEC | 122 | 32 (26) | 25 (54) | 6 (35) | 1 (2) |
| CR-AC | 263 | 52 (20) | 30 (34) | 17 (17) | 5 (7) |
| p-value^a^ |  | 0.153 | **0.021** | 0.072 | 0.216 |
| ***KRAS* mutated** |  |  |  |  |  |
| CR-NEC | 122 | 42 (34) | 15 (33) | 4 (23) | 23 (39) |
| CR-AC | 257 | 117 (45) | 45 (51) | 40 (40) | 30 (45) |
| p-value^a^ |  | **0.041** | **0.040** | 0.280 | 0.465 |
| ***APC* mutated*** |  |  |  |  |  |
| CR-NEC | 101 | 32 (32) | 8 (20) | 5 (38) | 19 (39) |
| CR-AC | 234 | 82 (35) | 29 (35) | 30 (33) | 22 (38) |
| p-value^a^ |  | 0.551 | 0.106 | 0.715 | 0.929 |
| ***TP53* mutated*** |  |  |  |  |  |
| CR-NEC | 101 | 60 (59) | 25 (64) | 6 (46) | 29 (59) |
| CR-AC | 234 | 126 (54) | 43 (52) | 46 (51) | 34 (58.6) |
| p-value^a^ |  | 0.347 | 0.202 | 0.738 | 0.953 |
| ***Rb1* mutated*** |  |  |  |  |  |
| CR-NEC | 101 | 8 (8) | 3 (8) | 0 | 5 (10) |
| CR-AC | 234 | 6 (2) | 4 (5) | 1 (1) | 1 (2) |
| p-value^a^ |  | **0.024** | 0.679 | 1 | 0.091 |
| **MSI** |  |  |  |  |  |
| CR-NEC | 107 | 4 (4) | 2 (4) | 0 | 2 (4) |
| CR-AC | 262 | 16 (6) | 14 (16) | 1 (1) | 1 (1) |
| p-value^a^ |  | 0.454 | 0.088 | 1 | 0.565 |

^a^For the comparison of mutation frequency Fischer’s exact test was used when sample size was < 5, otherwise Chi-Square Test was applied. Bold values indicate statistical significance (*p*<.05).

*Limited to coverage of the 46 gene hotspot panel applied for the CR-AC cohort. The number of CR-NEC mutations not covered by the hotspot panel: *TP53* n=4, *APC* n=17, *RB1* n=7.

# **Supplementary figures**

**Figure S1** Patient selection for the metastatic colorectal neuroendocrine carcinoma (CR-NEC) and the metastatic colorectal adenocarcinoma (CR-AC) cohort


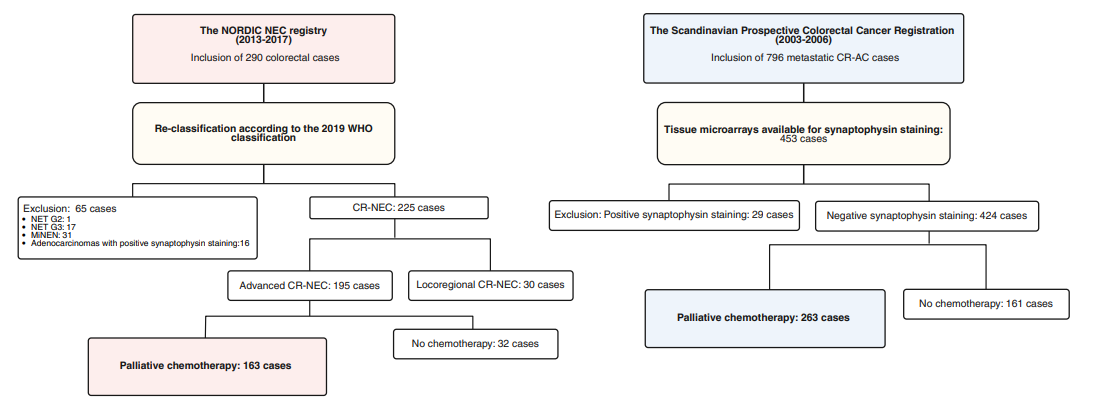


**Figure S2A** PFS in CR-NEC receiving first-line chemotherapy according to primary site


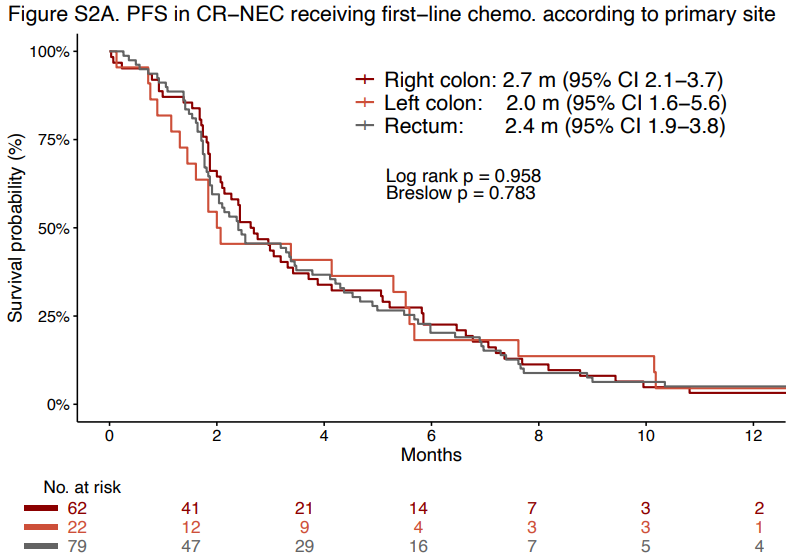


**Figure S2B** OS in CR-NEC receiving first-line chemotherapy according to primary site

**
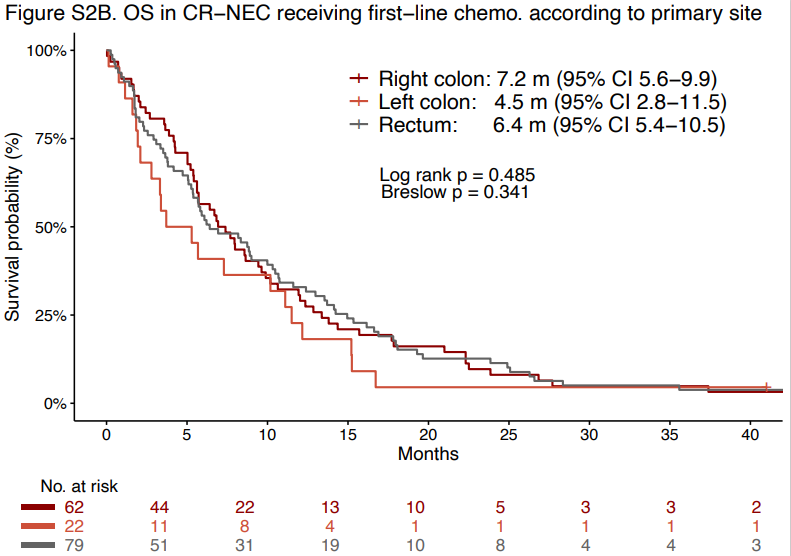
**

**Figure S3** PFS in CR-NEC receiving first-line chemotherapy according to performance status

**
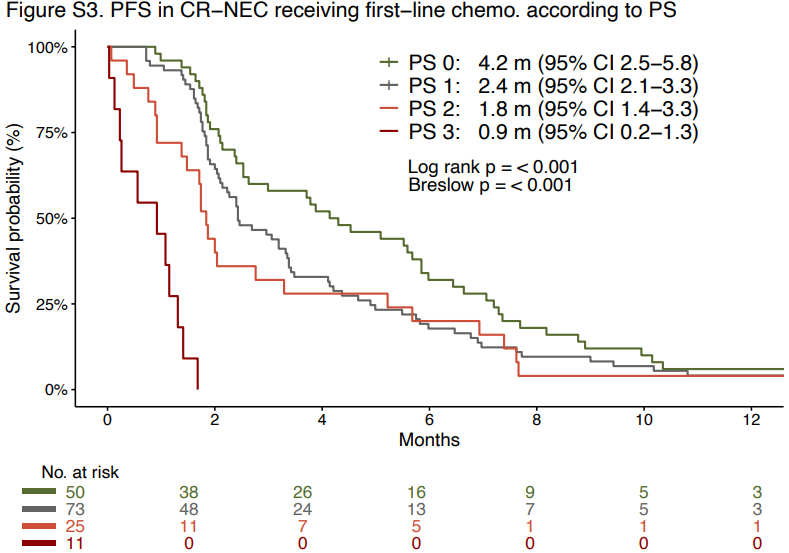
**

**Figure S4** Final multivariable model for survival following first-line chemotherapy in CR-NEC

**
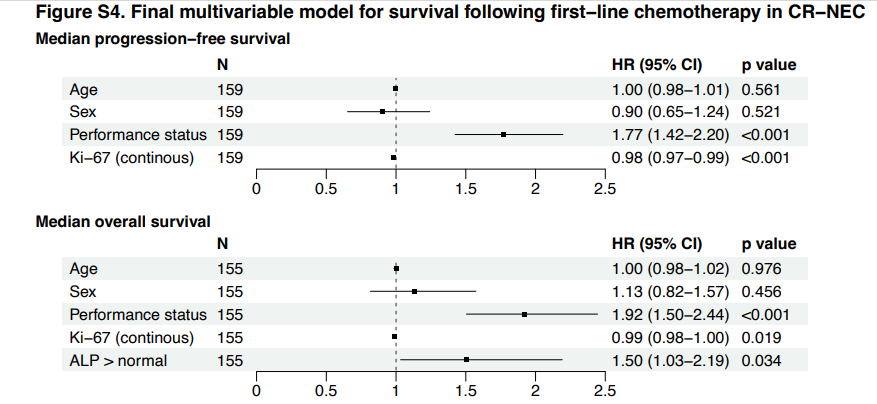
**

# **References**

1. Venizelos A, Elvebakken H, Perren A, Nikolaienko O, Deng W, Lothe IMB, Couvelard A, Hjortland GO, Sundlov A, Svensson J, Garresori H, Kersten C, Hofsli E, Detlefsen S, Krogh M, Sorbye H, Knappskog S. The molecular characteristics of high-grade gastroenteropancreatic neuroendocrine neoplasms. *Endocr Relat Cancer*. Nov 11 2021;29(1):1–14. doi:10.1530/ERC-21-0152

2. Yates LR, Gerstung M, Knappskog S, Desmedt C, Gundem G, Van Loo P, Aas T, Alexandrov LB, Larsimont D, Davies H, Li Y, Ju YS, Ramakrishna M, Haugland HK, Lilleng PK, Nik-Zainal S, McLaren S, Butler A, Martin S, Glodzik D, Menzies A, Raine K, Hinton J, Jones D, Mudie LJ, Jiang B, Vincent D, Greene-Colozzi A, Adnet PY, Fatima A, Maetens M, Ignatiadis M, Stratton MR, Sotiriou C, Richardson AL, Lonning PE, Wedge DC, Campbell PJ. Subclonal diversification of primary breast cancer revealed by multiregion sequencing. *Nat Med*. Jul 2015;21(7):751–9. doi:10.1038/nm.3886

3. Sorbye H, Dragomir A, Sundstrom M, Pfeiffer P, Thunberg U, Bergfors M, Aasebo K, Eide GE, Ponten F, Qvortrup C, Glimelius B. High BRAF Mutation Frequency and Marked Survival Differences in Subgroups According to KRAS/BRAF Mutation Status and Tumor Tissue Availability in a Prospective Population-Based Metastatic Colorectal Cancer Cohort. *PLoS One*. 2015;10(6):e0131046. doi:10.1371/journal.pone.0131046

4. Nunes L, Aasebo K, Mathot L, Ljungstrom V, Edqvist PH, Sundstrom M, Dragomir A, Pfeiffer P, Ameur A, Ponten F, Mezheyeuski A, Sorbye H, Sjoblom T, Glimelius B. Molecular characterization of a large unselected cohort of metastatic colorectal cancers in relation to primary tumor location, rare metastatic sites and prognosis. *Acta Oncol*. Apr 2020;59(4):417–426. doi:10.1080/0284186X.2019.1711169

5. Aasebo KO, Dragomir A, Sundstrom M, Mezheyeuski A, Edqvist PH, Eide GE, Ponten F, Pfeiffer P, Glimelius B, Sorbye H. Consequences of a high incidence of microsatellite instability and BRAF-mutated tumors: A population-based cohort of metastatic colorectal cancer patients. *Cancer Med*. Jul 2019;8(7):3623–3635. doi:10.1002/cam4.2205
